# An EBNA3C-deleted Epstein-Barr virus (EBV) mutant causes B-cell lymphomas with delayed onset in a cord blood-humanized mouse model

**DOI:** 10.1371/journal.ppat.1007221

**Published:** 2018-08-20

**Authors:** James C. Romero-Masters, Makoto Ohashi, Reza Djavadian, Mark R. Eichelberg, Mitch Hayes, Jillian A. Bristol, Shidong Ma, Erik A. Ranheim, Jenny Gumperz, Eric C. Johannsen, Shannon C. Kenney

**Affiliations:** 1 Department of Oncology, School of Medicine and Public Health, University of Wisconsin-Madison, Madison, Wisconsin, United States of America; 2 Department of Pathology and Laboratory Medicine, School of Medicine and Public Health, University of Wisconsin-Madison, Madison, Wisconsin, United States of America; 3 Department of Medicine, School of Medicine and Public Health, University of Wisconsin-Madison, Madison, Wisconsin, United States of America; 4 Department of Medical Microbiology and Immunology, School of Medicine and Public Health, University of Wisconsin-Madison, Madison, Wisconsin, United States of America; Baylor College of Medicine, UNITED STATES

## Abstract

EBV causes human B-cell lymphomas and transforms B cells *in vitro*. EBNA3C, an EBV protein expressed in latently-infected cells, is required for EBV transformation of B cells *in vitro*. While EBNA3C undoubtedly plays a key role in allowing EBV to successfully infect B cells, many EBV+ lymphomas do not express this protein, suggesting that cellular mutations and/or signaling pathways may obviate the need for EBNA3C *in vivo* under certain conditions. EBNA3C collaborates with EBNA3A to repress expression of the CDKN2A-encoded tumor suppressors, p16 and p14, and EBNA3C-deleted EBV transforms B cells containing a p16 germline mutation *in vitro*. Here we have examined the phenotype of an EBNAC-deleted virus (Δ3C EBV) in a cord blood-humanized mouse model (CBH). We found that the Δ3C virus induced fewer lymphomas (occurring with a delayed onset) in comparison to the wild-type (WT) control virus, although a subset (10/26) of Δ3C-infected CBH mice eventually developed invasive diffuse large B cell lymphomas with type III latency. Both WT and Δ3C viruses induced B-cell lymphomas with restricted B-cell populations and heterogeneous T-cell infiltration. In comparison to WT-infected tumors, Δ3C-infected tumors had greatly increased p16 levels, and RNA-seq analysis revealed a decrease in E2F target gene expression. However, we found that Δ3C-infected tumors expressed c-Myc and cyclin E at similar levels compared to WT-infected tumors, allowing cells to at least partially bypass p16-mediated cell cycle inhibition. The anti-apoptotic proteins, BCL2 and IRF4, were expressed in Δ3C-infected tumors, likely helping cells avoid c-Myc-induced apoptosis. Unexpectedly, Δ3C-infected tumors had increased T-cell infiltration, increased expression of T-cell chemokines (CCL5, CCL20 and CCL22) and enhanced type I interferon response in comparison to WT tumors. Together, these results reveal that EBNA3C contributes to, but is not essential for, EBV-induced lymphomagenesis in CBH mice, and suggest potentially important immunologic roles of EBNA3C *in vivo*.

## Author summary

Epstein-Barr virus is associated with multiple human B-cell malignancies and transforms B cells *in vitro* into immortalized cell lines. The EBV protein, EBNA3C, inhibits expression of the tumor suppressor protein, p16, and is required for EBV transformation of B cells *in vitro*. Here, we show that an EBNA3C-deleted virus (Δ3C) has a reduced ability (in comparison to the wild-type control virus) to cause B-cell lymphomas in a cord blood-humanized mouse model, although a subset of infected animals develop lymphomas at later time points. These tumors had high-level expression of the cell cycle inhibitor, p16, yet also expressed the cell cycle activators, c-Myc and cyclin E. Tumors also expressed pro-survival proteins, including BCL2 and IRF4. Unexpectedly, Δ3C tumors had increased T-cell infiltration, enhanced expression of several different T-cell chemokines, and a RNA-seq signature suggestive of an increased type 1 Interferon response. These results reveal that EBNA3C promotes EBV-induced lymphomas in the cord blood-humanized mouse model (but is not absolutely required), and suggest that EBNA3C may have immune modulatory effects *in vivo*.

## Introduction

Epstein-Barr virus (EBV) is a human gamma-herpesvirus that infects 90% of the world’s adult population [1]. EBV establishes a lifelong infection in the memory B cell compartment, and periodically reactivates to the lytic form of viral infection when B cells are stimulated by antigen and/or differentiate into plasma cells [1–3]. In addition to causing the clinical syndrome, infectious mononucleosis, EBV is associated with multiple human malignancies of B-cell and epithelial origin, including Burkitt lymphoma, Hodgkin lymphoma, diffuse large B cell lymphoma (DLBCL), post-transplant lymphoproliferative disease (PTLD), nasopharyngeal carcinoma and gastric carcinoma [4–6]. All EBV-associated malignancies are latently infected with EBV.

EBV infection of B cells *in vitro* results in immortalized lymphoblastoid cell lines (LCLs) that produce lymphomas when injected into immunosuppressed mice, and much of our current understanding of the transforming functions of latent EBV proteins is derived from LCL models. However, while at least three different types of viral latency (types I, II and III) can occur in B cells during EBV infection *in vivo*, type III latency (in which all 9 latency proteins are expressed) is the only latency type that transforms B-cells *in vitro* into LCLs [6]. EBV-induced transformation of B cells *in vitro* is largely driven by the two major EBV-encoded oncoproteins, EBNA2 and LMP1 [7]. EBNA2 is a strong transcriptional activator that turns on the viral promoters driving latent viral gene transcription during type III latency, and also upregulates cellular genes such as c-Myc and Cyclin D2 that induce LCL proliferation [8–11]. EBNA2 directly interacts with the cellular RBPJK DNA binding protein (that normally mediates intracellular Notch binding) to produce a Notch-like signal [12,13]. The other major EBV oncoprotein, LMP1, is a transmembrane protein that mimics the effect of constitutive CD40 signaling, which results in NF-kB activation [14–18]. The NF-kB pathway induces survival and proliferation of B cells and is activated in many human B-cell lymphomas [19].

In addition to EBNA2, three other latent EBV nuclear proteins, EBNA3A, EBNA3B, and EBNA3C, also interact with RBPJK in LCLs, and both EBNA3A and EBNA3C are required for LCL generation *in vitro* [6]. The EBNA3 proteins regulate distinct but overlapping sets of cellular genes *in vitro* [20–25]. EBNA3A and EBNA3C collaborate to induce repression of the CDKN2A-encoded tumor suppressors, p16INK4a (p16) and p14ARF (p14), an effect associated with increased H3K27me3 modification at the CDKN2A promoter [26–28]. Expression of the CDKN2A locus is activated by many cellular and viral oncoproteins, and the p14/p16 proteins encoded by this locus inhibit the ability of oncoproteins to transform cells by preventing cell cycle progression and inducing cellular senescence [29–34]. Turn-off of EBNA3C expression in established LCLs *in vitro* results in high level p16 expression and inhibits cellular proliferation [35,36]. Importantly, EBNA3C-deleted EBV can transform B cells containing a germline mutation inactivating p16 into LCLs *in vitro* [27]. Thus, the most essential role of EBNA3C in EBV-induced transformation of B cells *in vitro* is inhibiting oncogene-induced expression of p16.

Numerous other proposed functions of EBNA3C may also contribute to the viability of EBV-infected B cells *in vivo*. In addition to preventing p16-induced cellular senescence, EBNA3C attenuates an anti-proliferative DNA damage response that inhibits EBV transformation of primary B lymphocytes *in vitro* [37], and upregulates expression of the cellular AICDA gene [38]. AICDA encodes the activation-induced cytosine deaminase protein (AID), which is required for immunoglobulin class switching and somatic hyper-mutation of B cells [39], and is thought to promote the c-Myc translocations that drive Burkitt lymphomas [40]. EBNA3C has also been implicated in preventing c-Myc induced apoptosis through inhibition of BIM activation in Burkitt lymphoma cell lines [34,41,42]. In addition, EBNA3C is reported to enhance stability of the B-cell survival protein, IRF4 [43], and to inhibit p53 function *in vitro* [44]. Furthermore, although EBNA3 proteins interact with an RBPJK domain which is different from the domain that interacts with EBNA2, they compete for RBPJK binding and limit EBNA2 activation of certain promoters [20,45–48]. Thus, EBNA3C could potentially affect EBV transformation *in vivo* by modulating EBNA2 function. EBNA3C also reduces expression of several different T-cell chemokines when expressed in the an EBV-negative Burkitt cell line *in vitro* [49], and could potentially inhibit T-cell infiltration of tumors via this mechanism. Nevertheless, since many EBV+ human lymphomas do not express EBNA3C, it may be less important for growth of EBV+ lymphomas *in vivo*, suggesting that cellular mutations and/or signaling pathways may obviate the need for EBNA3C. In addition, the epigenetic modifications induced by EBNA3C during early infection may decrease the need for continued expression of EBNA3C at later time points [50].

Humanized mouse models provide an opportunity to study EBV infection *in vivo* in an environment containing human immune cells, and can lead to novel insights regarding the roles of EBV latency proteins. We previously used a humanized mouse model to show that an LMP1-deleted EBV mutant that is non-transforming *in vitro* can cause lymphomas *in vivo* due to the ability of human CD40 ligand-expressing CD4 T cells to provide an alternative source of CD40 signaling [51,52]. Here, we have used a cord blood-humanized mouse model to examine the phenotype of an EBNA3C-deleted EBV mutant *in vivo*. We find that this mutant is partially impaired for the ability to induce lymphomas in this model, although a subset of infected animals develop DLBCLs with delayed kinetics. By comparing the phenotypes of lymphomas containing wild-type (WT) versus EBNA3C-deleted viruses in this model, we have confirmed some previously reported *in vitro* functions of EBNA3C (inhibition of p16 expression and activation of AICDA expression) and have discovered potentially new functions (including inhibition of T cell infiltration and type 1 interferon signaling). These results reveal that EBNA3C is important but not essential for the development of EBV-induced lymphomas in cord blood-humanized mice.

## Results

### EBNA3C-deleted EBV (Δ3C) causes lymphomas in a cord blood-humanized mouse model, but with a reduced efficiency and delayed onset compared to WT EBV

To examine the effect of EBNA3C loss on the ability of EBV to induce lymphomas in humanized mice *in vivo*, we inactivated the EBNA3C gene in the B95.8 EBV bacmid p2089 by inserting a stop codon near the start of the EBNA3C protein as described in the methods section. A revertant mutant was also constructed. 293 cell lines stably infected with either mutant and revertant bacmids were selected for as described in the methods. Infectious virion particles were produced by transfecting 293 cells with BZLF1 (Z), BRLF1 (R) and BALF4 (GP110) expression vectors. Virus was then concentrated and titered using the “green Raji cell assay” as previously described [53].

The cord blood-humanized mouse model, which we previously showed supports the development of lymphomas induced by a LMP1-deleted EBV mutant [51,52], was used to compare lymphomagenesis outcomes from wild-type (WT) versus EBNA3C-deleted (Δ3C) EBV *in vivo*. CD34-depleted human umbilical cord blood was either mock infected or infected with 5,000 infectious particles (green Raji units) of WT or Δ3C EBV for 1.5 hours *in vitro* and then injected intraperitoneally (i.p.) into NSG (NOD/LtSz-scid/IL2Rnull) mice. Approximately 10 million nucleated cord blood cells (containing a mixture of B cells, T cells, NK cells, monocytes and dendritic cells) were injected into each mouse. In each experiment, WT and mutant virus infection was performed using the same cord blood sample, and experiments were repeated multiple times using different cord blood samples. We have previously shown that WT (B95.8 bacmid) EBV induces lymphomas in nearly all animals in this model, whereas mock-infected animals do not develop lymphomas [52]. Animals were sacrificed when they showed signs of illness, or otherwise euthanized at day 90 after cord blood cell injection. Animals were considered to have EBV-infected lymphomas if they had large numbers of EBNA2- (or EBNA1-) positive B cells invading one or more organs.

**Fig 1:**
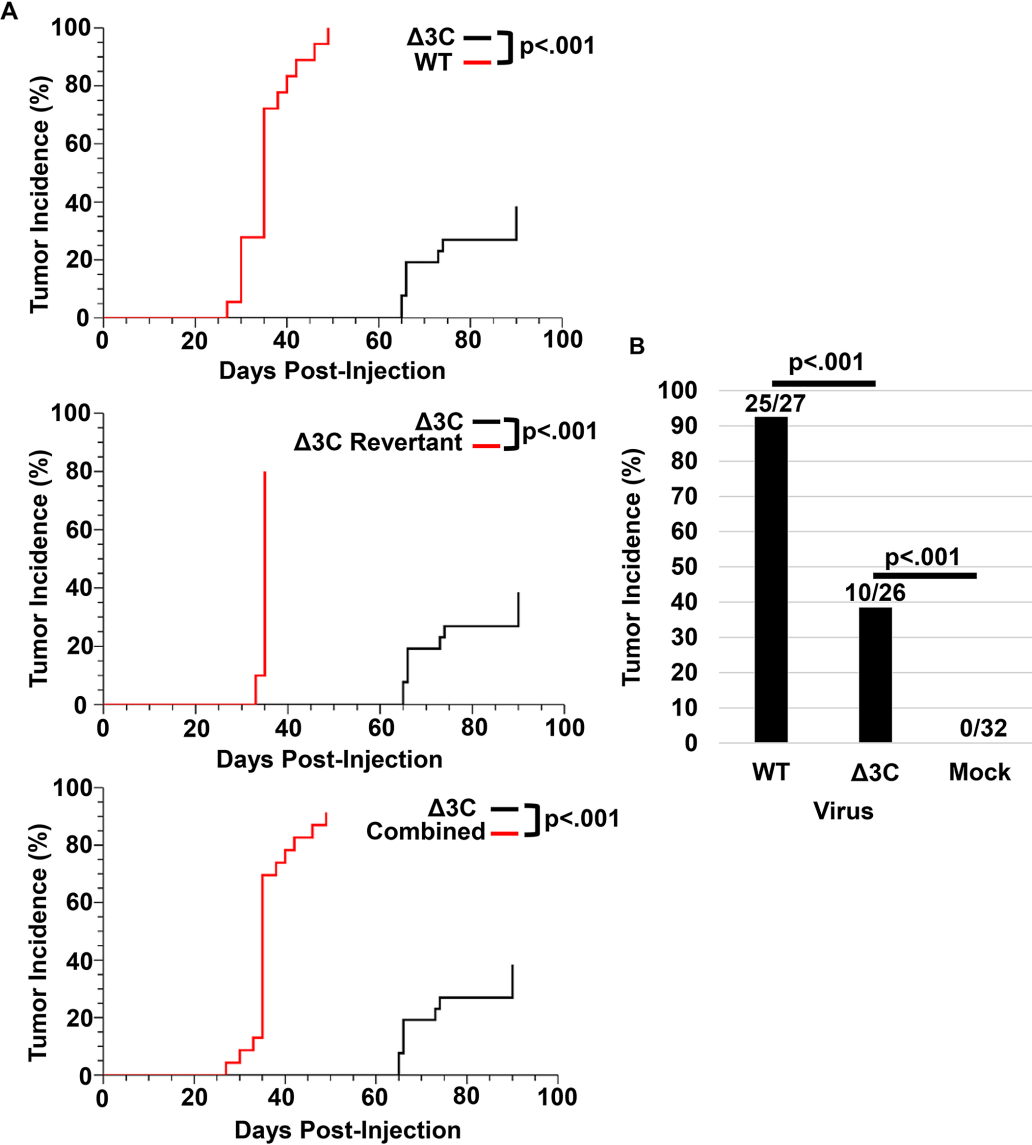
EBNA3C-mutant virus (Δ3C) causes lymphomas at reduced efficiency, and with a delayed-onset, in the cord blood-humanized (CBH) mouse model. (A). The rate and incidence of tumors in animals infected with WT versus Δ3C viruses are shown (upper panel). The rate and incidence of tumors in animals infected with revertant virus versus Δ3C viruses are shown (middle panel). The rate and incidence of tumors in animals infected with either WT or revertant viruses, versus Δ3C virus, are shown (lower panel). A log-rank statistical test was performed to determine statistical significance for the Kaplan-Meier curve analysis. (B) The frequency of EBV-positive tumors in animals infected with wild-type or revertant control viruses (WT), the Δ3C virus, or mock-infected animals, is shown. A two-sided Bernard’s exact test was performed to determine statistical significance of any differences in tumor frequency.

As shown in Fig 1, the Δ3C-infected animals required a significantly longer time to develop tumors (66–90 days) compared to WT-infected animals (28–35 days). Since the revertant virus-infected lymphomas formed at a similar time-frame, and with a similar efficiency, as the WT virus-induced lymphomas (Fig 1A), results from WT and revertant EBV infection were aggregated (and referred to as “WT” EBV) for the rest of the analyses shown in this paper. In addition to producing lymphomas at a delayed time point relative to WT EBV, Δ3C EBV-infected animals had significantly fewer lymphomas in the cord blood-humanized mouse model (10/26 infected animals) in comparison to WT EBV (25/27 infected animals) (Fig 1B). We confirmed that lymphomas induced by the EBNA3C-mutant virus contained the expected mutation (insertion of a stop codon at residue 2 in the EBNA3C protein) by sequencing viral DNA isolated from lymphomas on formalin-fixed paraffin-embedded (FFPE) slides infected with WT or Δ3C viruses (S1 Fig). These results confirm that EBNA3C expression contributes to efficient EBV-induced lymphoma formation in the cord blood-humanized mouse model. Nevertheless, since the Δ3C-infected animals developed significantly more tumors than mock-infected animals (0/32), loss of EBNA3C expression does not completely prevent EBV from inducing lymphomas in cord blood-humanized mice. Interestingly, the great of majority of tumor-free Δ3C-infected animals had no evidence of persistent viral infection (EBER+ cells by *in situ* hybridization) at the time of euthanasia. The unexpected ability of the Δ3C virus to induce lymphomas in a subset of animals in this model allowed us to compare the characteristics of lymphomas infected with the two different types of viruses.

### Δ3C EBV induces aggressive DLBCLs with high level p16 expression

**Fig 2:**
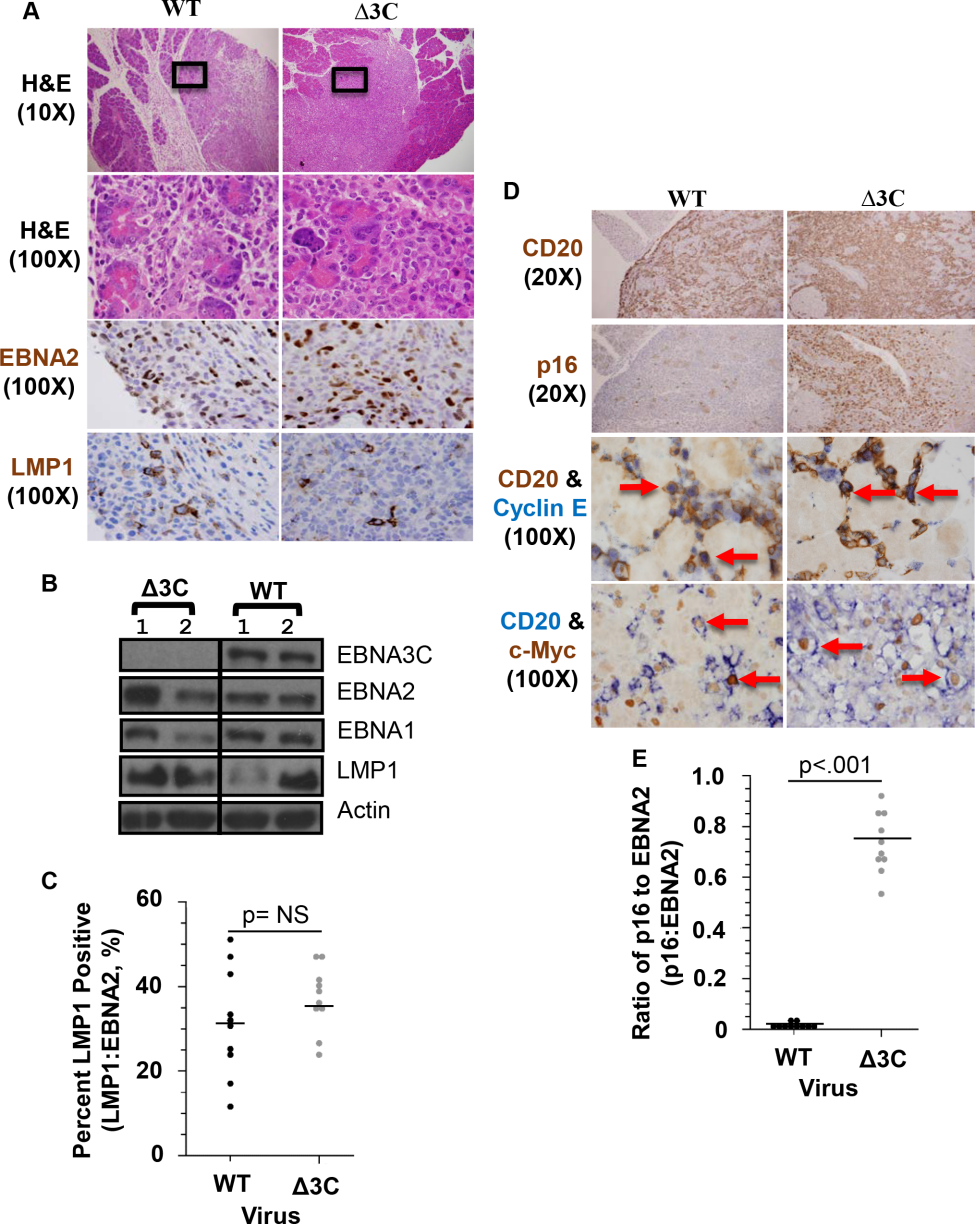
Δ3C virus causes DLBCLS with type III viral latency that express high levels of p16, but still express cyclin E and c-Myc. (A) Lymphomas invading the pancreas, harvested from CBH mice injected with WT control or Δ3C viruses, are shown. Tissues were formalin-fixed and paraffin-embedded, and then subjected to H&E stain, and IHC staining for EBNA2 (EBV latent protein) and LMP1 (EBV latent protein) on adjacent slides as indicated (WT: SK999 and Δ3C: SK1005). (B) Protein extracts derived from lymphomas infected with WT (SK449R and SK447R) or Δ3C viruses (SK449LR and SK447U) were used to perform immunoblots to detect EBNA3C, EBNA2, EBNA1, LMP1 and actin as indicated. (C) The percent LMP1-positive cells were determined by comparing the total number of LMP1-positive cells versus the total number of EBNA2-positive cells on adjacent slides in 10 tumors infected with either the WT or Δ3C viruses. Wilcoxon rank-sum statistical test (two-sided) was performed. (D) IHC single staining was performed using antibodies against CD20 (B-cell marker), or p16 on adjacent slides (WT: SK999 and Δ3C: SK1005), and co-staining IHC studies were performed using antibodies against cyclin E (purple) and CD20 (brown) (WT: SK1189 and Δ3C: SK1183), or c-Myc (brown) and CD20 (purple) (WT: SK1331 and Δ3C: SK1348), as indicated. Examples of co-staining cells are indicated with arrows. (E) Quantification of the ratio of EBNA2-positive to p16-positive cells in 10 different WT- and Δ3C-infected animals is shown. Wilcoxon rank-sum test was performed. The EBV infected animals used in each image are indicated for each figure and are further detailed in S3 Table.

To compare the phenotypes of lymphomas induced with the WT versus Δ3C viruses, tumors were stained with hematoxylin and eosin (H&E). Animals infected with either the WT or Δ3C viruses developed aggressive DLBCLs that invaded various organs (particularly the pancreas, as shown in Fig 2A, as well as the liver, gall bladder, abdominal lymph nodes and less commonly the spleen) (S1 Table). There was no consistent difference in the sizes of lymphomas induced by the WT versus Δ3C viruses. In each case, the tumors consisted of sheet-like expansions of morphologically atypical large cells with prominent nucleoli and irregular nuclei. The morphology ranged from immunoblastic to frankly anaplastic between different mice, but no consistent morphologic differences were noted between WT virus- versus Δ3C virus- induced lymphomas. To determine if DLBCLs have type III EBV latency, immunohistochemistry (IHC) staining and immunoblot analysis was performed using antibodies that detect either the EBNA2 or LMP1 proteins. Tumors infected with either the WT or Δ3C viruses expressed both EBNA2 and LMP1 (Figs 2A, 2B and S2), indicating that they each have type III latency. To determine if loss of EBNA3C affects the ability of EBNA2 to activate the LMP1 promoter (which has previously been reported to be synergistically activated by the EBNA2/EBNA3C combination *in vitro* [54,55]), the relative number of LMP1-positive cells versus EBNA2-positive cells was quantitated. As shown in Fig 2C, WT- and Δ3C-infected lymphomas had a similar number of LMP1-positive cells relative to EBNA2-positive cells (Fig 2C).

To assess whether WT- and Δ3C-infected lymphomas contain similar numbers of EBV-infected B cells, we performed EBER, EBNA1, and CD20 stains on adjacent slides and quantified the ratio of cells expressing each EBV marker to the number of CD20 (a B-cell marker)-positive cells. The number of cells expressing each EBV marker was similar to the number of CD20 positive cells in each tumor type (S3 Fig and S2 Table). These results indicate that WT and EBNA3C-deleted viruses induce lymphomas containing a similar number of EBV-infected B cells.

Given the ability of EBNA3C to inhibit p16 expression in LCLs, and the essential role that inhibition of p16 expression plays for LCL generation *in vitro* [36], we next asked if p16 expression is altered in Δ3C virus-infected, versus WT virus-infected, lymphomas. As shown in Fig 2D and 2E, IHC staining of tumors using an antibody directed against p16 revealed a marked increase in p16 expression in the Δ3C virus-induced lymphomas. This result confirms that EBNA3C significantly inhibits p16 expression in EBV-infected lymphoma cells in cord blood-humanized mice.

Although high-level p16 expression normally halts cellular proliferation by inhibiting the ability of cyclin D/CDK4/6 complexes to phosphorylate and inactivate pRb [33], human melanomas and DLBCLs can escape the effect of p16 by inducing cyclin E expression [56,57]. High-level c-Myc expression (which activates cyclin E expression) can also bypass the p16 effect [58]. We therefore performed IHC using antibodies against CD20 (a B-cell marker), cyclin E, and c-Myc to compare the levels of cyclin E and c-Myc in CD20+ B cells of Δ3C virus-infected versus WT virus-infected lymphomas (Fig 2D). The Δ3C virus-infected lymphoma cells expressed both cyclin E and c-Myc at levels similar to that found in the WT virus-infected cells. This result suggests that Δ3C virus-infected lymphomas can bypass the cell cycle inhibitory effect of p16 by expressing cyclin E and/or c-Myc.

### Δ3C-induced lymphomas have low expression of the pro-apoptotic protein, BIM, and express the anti-apoptotic proteins, BCL2 and IRF4

**Fig 3:**
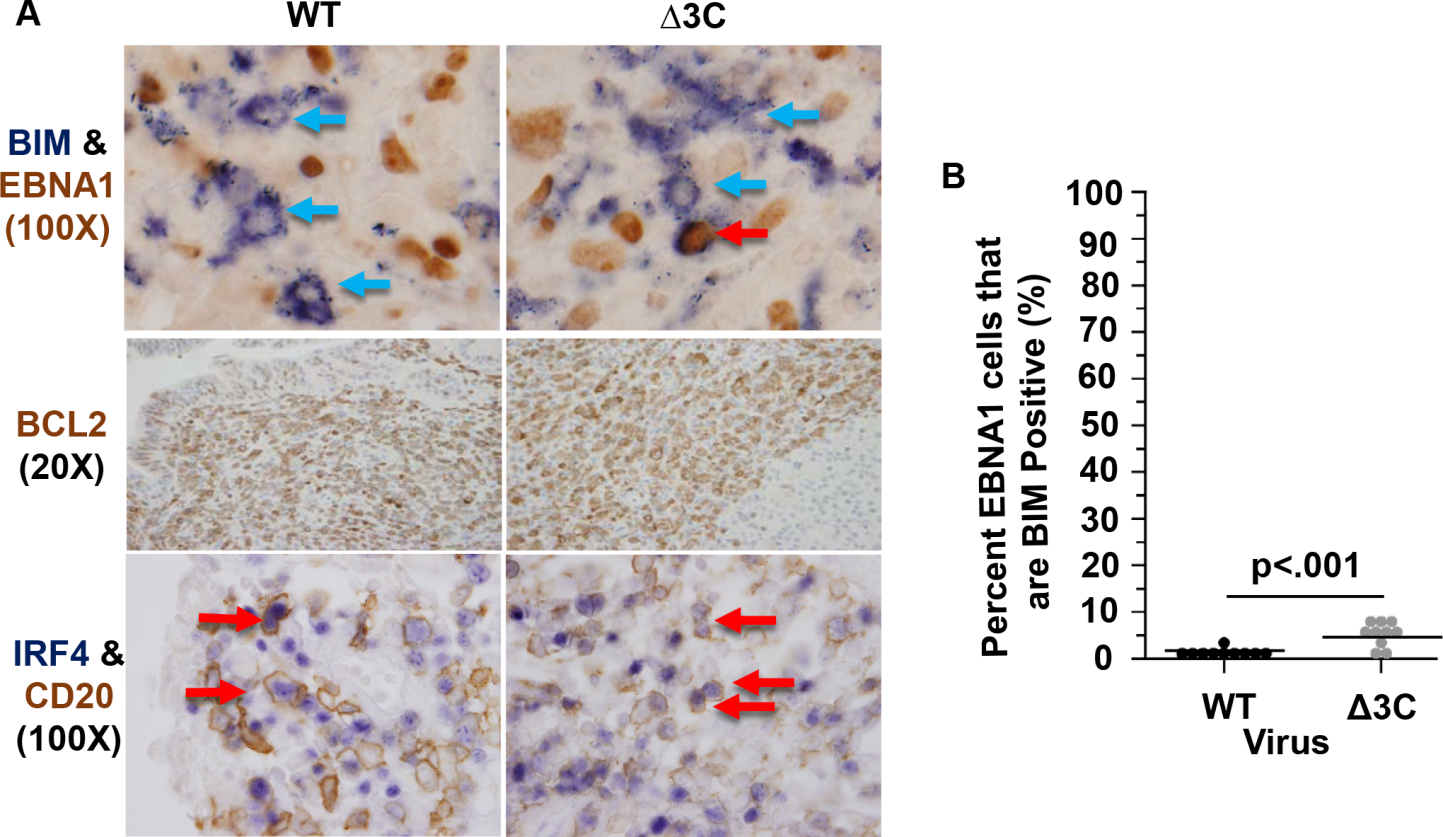
Δ3C-induced lymphomas express low levels of the pro-apoptotic protein, BIM, and express the pro-survival proteins, BCL2 and IRF4. (A) IHC co-staining was performed for BIM (purple) and EBNA1 (brown) (WT: SK1331 and Δ3C: SK1348), or CD20 (brown) and IRF4 (purple) (WT: SK1335 and Δ3C: SK1340), as indicated. Representative BIM1/EBNA1 or IRF4/CD20 co-staining is indicated with red arrows and representative BIM+ EBNA1- cells indicated with blue. An example of a BIM+/EBNA1+ cell in a Δ3C-induced lymphoma is indicated. Single staining was performed for BCL2 (WT: SK1193 and Δ3C: SK1183). (B) The number of BIM+, EBNA1+ co-staining cells compared to the total number of EBNA1-positive cells was quantitated in each tumor type. Wilcoxon rank-sum test was performed.

Since the pro-apoptotic protein, BIM, is an important tumor suppressor for c-Myc-induced lymphomas [34,41], and EBNA3C inhibits expression of BIM in Burkitt lymphoma cell lines *in vitro* [42], we performed IHC analysis using antibodies against the EBV latency protein, EBNA1, and BIM to compare BIM expression in WT virus- versus Δ3C virus-infected lymphoma cells. Consistent with previous results [42], BIM expression was significantly higher in the Δ3C virus-infected lymphoma cells versus the WT virus-infected cells (Fig 3A and 3B). Nevertheless, we found that the majority of EBNA1-positive cells did not co-express BIM in either tumor type (Fig 3A and 3B). Many BIM+ cells that did not co-stain for EBNA1 (presumably EBV-negative T cells) were also observed (Fig 3A). This result suggests that in addition to EBNA3C, other viral and/or cellular factors can suppress BIM expression in EBV-induced B-cell lymphomas with type III latency in the cord blood-humanized mouse model.

We also compared the levels of the anti-apoptotic proteins, BCL2 and IRF4, in the two tumor types. Both BCL2 and IRF4 expression are reported to be induced by NF-kB signaling [59–61], which is activated by LMP1. BCL2 inhibits apoptosis by inhibiting the pro-apoptotic proteins, BAX and BAK1 [62]. IRF4 is a surrogate immunohistochemical marker for the “activated B cell” subtype of DLBCLs and is also an essential survival factor for activated B cell lymphomas in humans [63,64]. EBNA3C has been shown to stabilize IRF4 expression *in vitro*, and regulates certain cellular genes in a complex with IRF4 [20,43]. IHC analysis using antibodies against BCL2, IRF4, and CD20 showed a similar level of both BCL2 and IRF4 in CD20 co-staining B cells in the WT virus- and Δ3C virus-induced tumors (Fig 3A). These results suggest that EBNA3C is not required for IRF4 or BCL2 expression in EBV-infected lymphomas. Nevertheless, we cannot exclude the possibility that the essential role of IRF4 in activated DLBCLs selects for EBV-independent IRF4 expression in Δ3C virus-induced tumors.

### Δ3C EBV-induced lymphomas have increased T cell infiltration compared to WT virus-induced lymphomas

**Fig 4:**
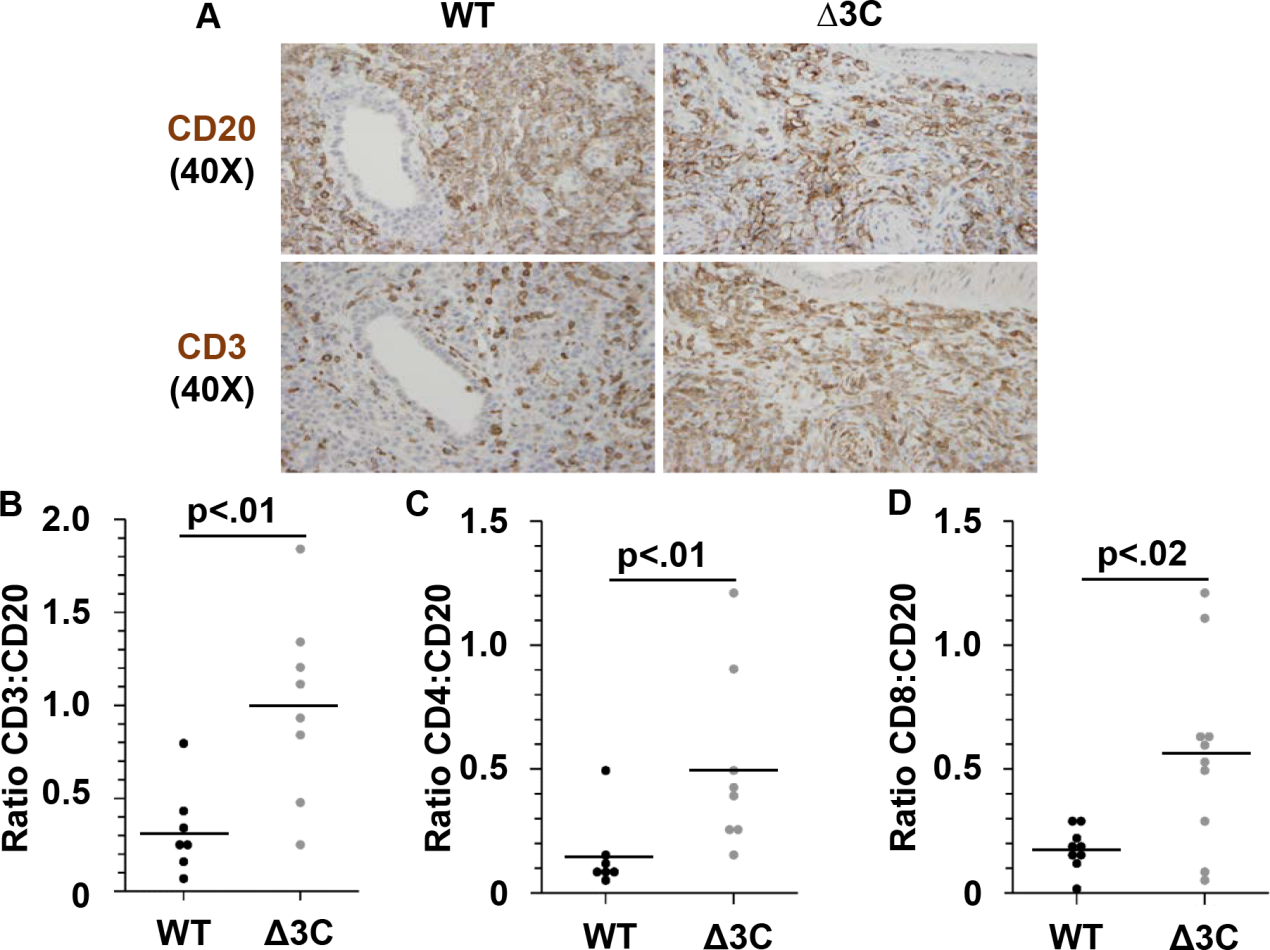
Δ3C virus-induced lymphomas have increased T-cell infiltration of both CD8+ and CD4+ T cells. (A) IHC staining was performed for CD20 (B-cell marker) and CD3 (total T-cell marker) on adjacent slides (WT: SK1189 and Δ3C: SK1192). Representative images are shown for WT- and Δ3C-infected animals. (B) Quantification of the ratio of CD3+ cells to CD20+ cells in at least 7 different tumors from each condition is shown. (C) Quantification of the ratio of CD4+ cells to CD20+ cells in at least 7 different tumors. (D) Quantification of the ratio of CD8+ cells to CD20+ cells in at least 9 different tumors. Wilcoxon rank-sum test was performed on all quantification analyses.

An EBV mutant deleted for the EBNA3B protein was previously shown to produce lymphomas with decreased T-cell infiltration (in comparison to WT virus) in humanized mice, possibly due to decreased expression of the T-cell chemokine, IP10, in B cells infected with this virus [65]. In contrast, EBNA3C reduces expression of several different chemokines when expressed in the EBV-negative BJAB Burkitt cell line *in vitro*, including the CCL3, CCL4, CXCL10 and CXCL11 chemokines, which can each attract T cells to infected tissues [49]. Thus, EBNA3C could potentially act to inhibit T-cell infiltration of EBV-infected lymphomas *in vivo*. To determine if lymphomas infected with the Δ3C EBV have altered T-cell infiltration in comparison to wild-type virus infected lymphomas in cord-blood humanized mice, we compared the number of CD3-positive cells to the number of CD20-positive cells in each tumor type. This analysis revealed that Δ3C-virus induced lymphomas had increased T-cell infiltration compared to the WT-induced lymphomas (Figs 4A, 4B and S4). While the absolute number of B cells per 40X field was similar between WT- and Δ3C-induced lymphomas (S4 Fig), the absolute number of T cells was increased. IHC analysis using antibodies against CD4 (helper T cells) and CD8 (cytotoxic T cells) showed a significant increase in both T-cell populations in the Δ3C-induced tumors compared to the WT-induced tumors (Fig 4C and 4D). These results suggest that EBNA3C expression, in contrast to EBNA3B expression, may be associated with reduced T-cell infiltration into EBV-induced lymphomas.

### WT and EBNA3C-deleted tumors are dominated by expansions of a limited number of individual B cells

To further examine expression of viral and cellular genes in WT virus-induced, versus Δ3C virus-induced, lymphomas, RNA was isolated from three different tumors infected with each virus type, and RNA-seq was performed. In this analysis, RNA sequences were derived from a mixture of EBV-infected tumor cells and infiltrating T cells; contaminating mouse cell genes were removed from the bioinformatics analysis as described in the methods.

**Fig 5:**
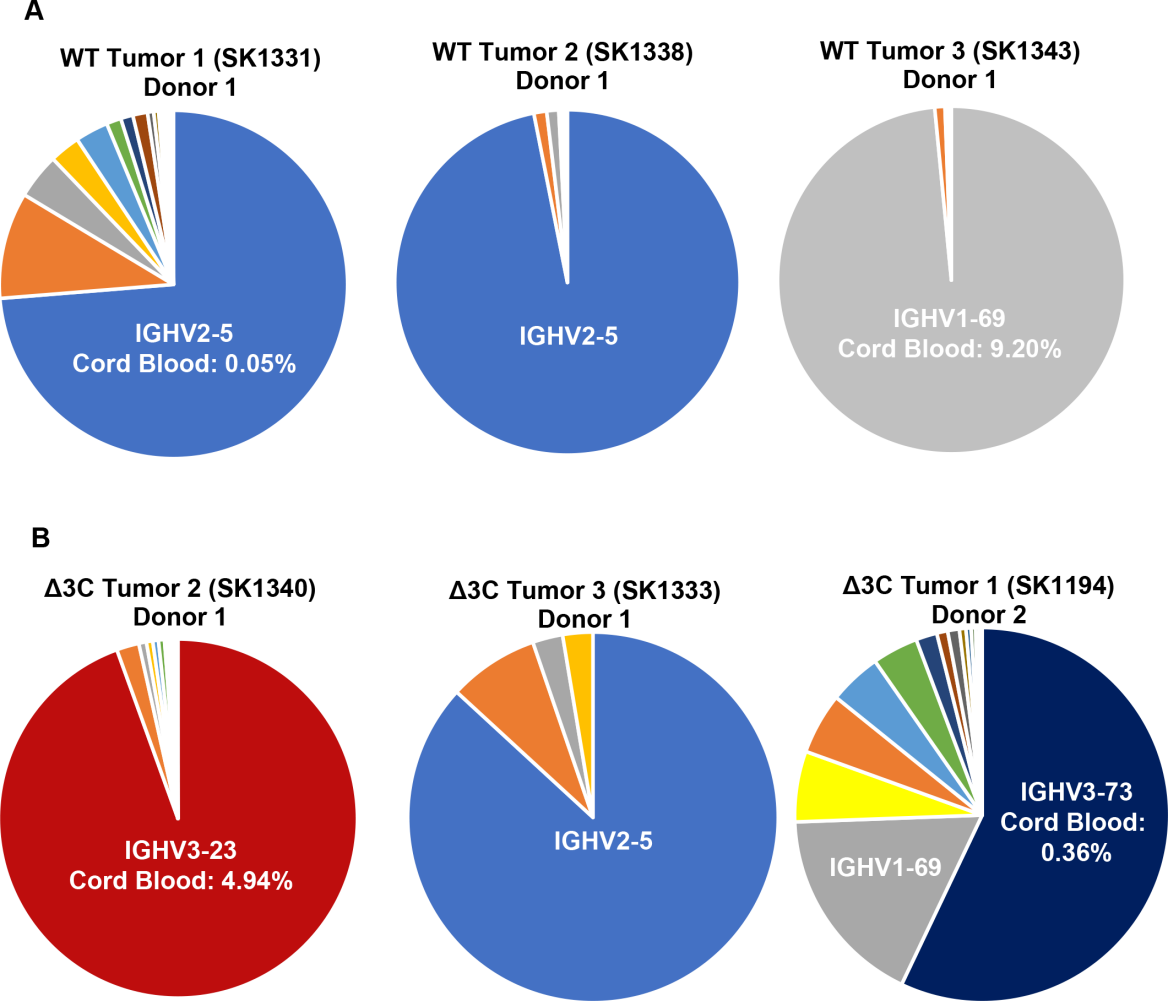
WT virus- and Δ3C virus-induced lymphomas are derived from a restricted B-cell population. (A-B) RNA-seq analysis was performed using RNA isolated from 3 different tumors infected with either the WT or Δ3C viruses. The relative frequency of the IGH transcripts containing various different IGHV genes is shown for each tumor; the frequency of the dominant IGHV genes in IGH transcripts of normal cord blood is also indicated.

To investigate the characteristics of the B cell populations of EBV-induced lymphomas infected with WT or Δ3C viruses, we compared the expression levels of the 40 human IGHV genes in each tumor as described in the methods. In most tumors (both WT- and Δ3C-infected), a single dominant IGHV gene was utilized (Fig 5). One of the three Δ3C-induced lymphomas are examined was composed of two different major clones, and a mixture of other IGHV sequences (Fig 5B). These results suggest that both WT EBV- and Δ3C-induced lymphomas are mainly derived from restricted expansions of a small initial number of EBV-infected B cells, rather than consisting of highly heterogeneous expansions of a broad selection of different EBV-infected B cells. Interestingly, 3 of the 6 tumors examined harbored the same dominant IGHV gene (IGHV2-5), and 2 of the tumors expressed the IGHV1-69 gene as a dominant clone (Fig 5). In comparison, IGH transcripts containing IGHV2-5 constitute only 0.05% of total IGH transcripts in normal cord blood, while transcripts containing IGHV1-69 constitute 9% of total IGH transcripts [66].

The CDR3 sequences of BCR clones in each tumor were analyzed as described in the methods. The major BCR clones in each tumor had distinct CDR3 sequences (S5 Fig). To determine if WT virus-infected, or Δ3C virus-infected lymphomas had undergone somatic hyper-mutation, the CDR3 sequences of dominant IGH clones were also analyzed using publicly available software (IMGT) as described in the methods. This analysis revealed that the CDR3 sequences had undergone little if any somatic hyper-mutation in either WT EBV- or Δ3C virus-infected lymphomas (S5 Fig). Together, these results indicate that the usage of certain variable heavy chain genes may be selected for during the development of EBV-derived B-cell tumors in the cord blood-humanized mouse model, but somatic hyper-mutation is unlikely to contribute to the formation of these tumors.

### WT virus- and Δ3C virus-infected tumors contain a heterogeneous T-cell infiltrates

**Fig 6:**
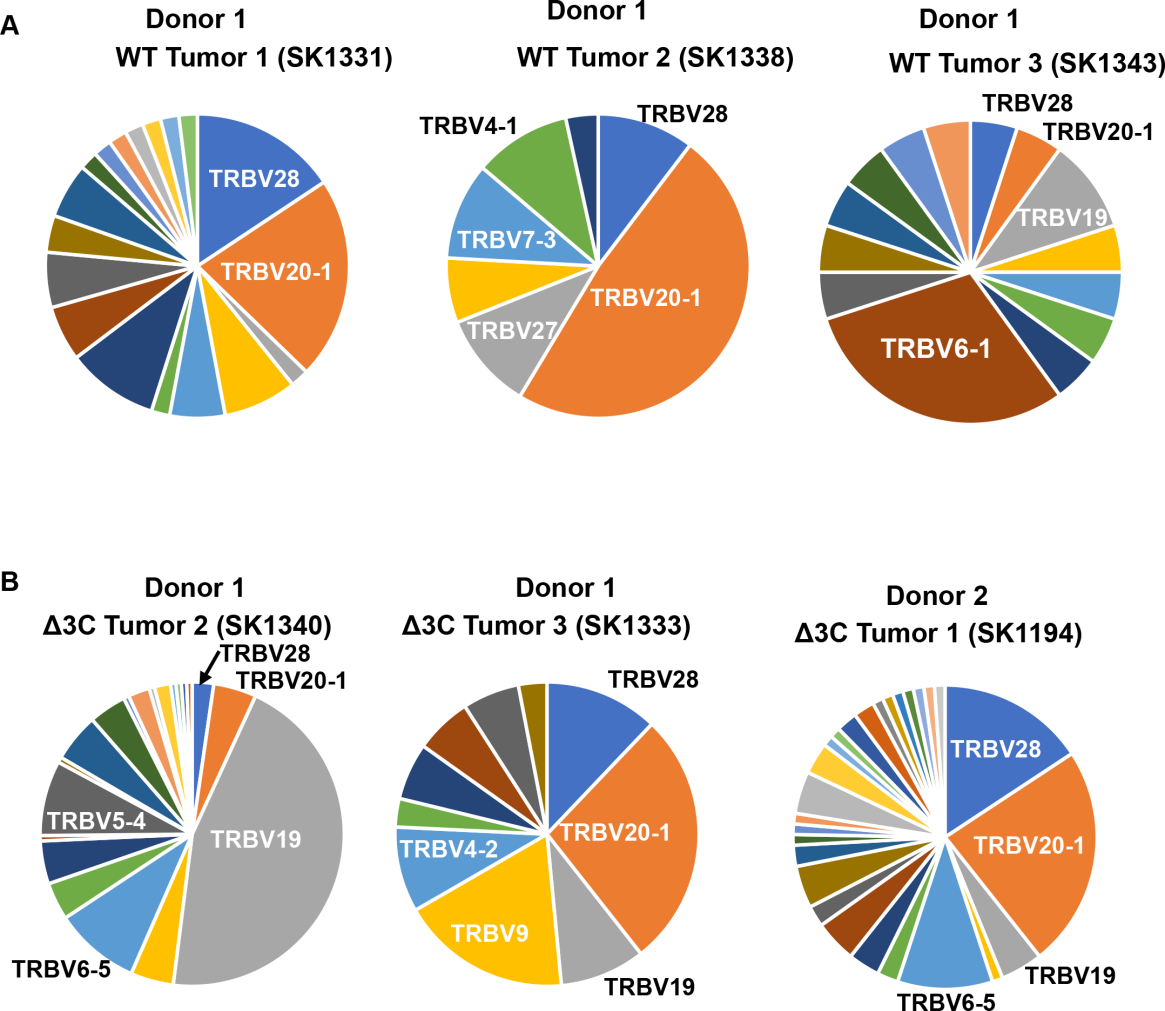
WT virus and Δ3C virus-induced lymphomas have a heterogeneousT cell response. (A-B) The frequency of the TRBV gene reads in the RNA-seq analysis of different tumors was determined by comparing the number of reads from each individual TRBV gene to the total number of TRBV gene reads.

To investigate the T cell infiltrates of EBV-induced lymphomas infected with the WT or Δ3C viruses, we compared the expression levels of the 42 different human TCR beta variable (TRBV) genes in each tumor (Fig 6). All tumors contained a variety of TCRs. CDR3 sequences of some TCR beta chains in each tumor were also determined by analysis of RNAseq results as described in the methods. A number of different CDR3 sequences were present, with no single CDR3 identified in multiple tumors (S4 Table). Together, the analysis of the IGH and TCR beta gene expression patterns indicate that EBV-induced tumors contain restricted B-cell populations, and heterogeneous T-cell populations.

### Analysis of EBV gene expression in Δ3C virus-infected versus WT virus-infected lymphomas

**Fig 7:**
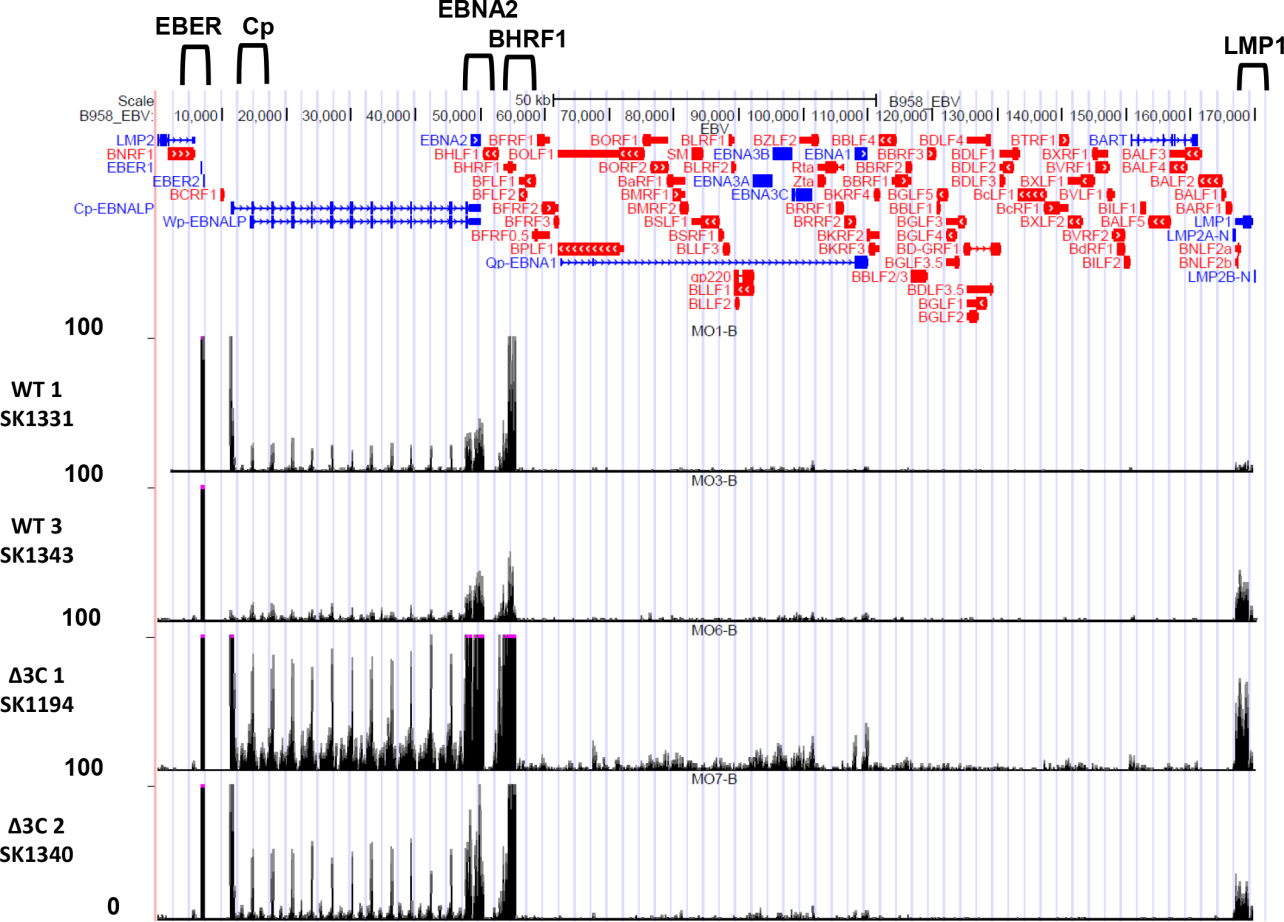
EBV genome mapping of RNAseq reads in Δ3C virus- and WT virus-induced lymphomas. RNA-seq reads were mapped to the EBV genomes of 2 different tumors infected with each virus type. The locations of the Cp promoter, and the EBER, EBNA2, BHRF1 and LMP1 transcripts are indicated above the EBV genome map.

**Fig 8:**
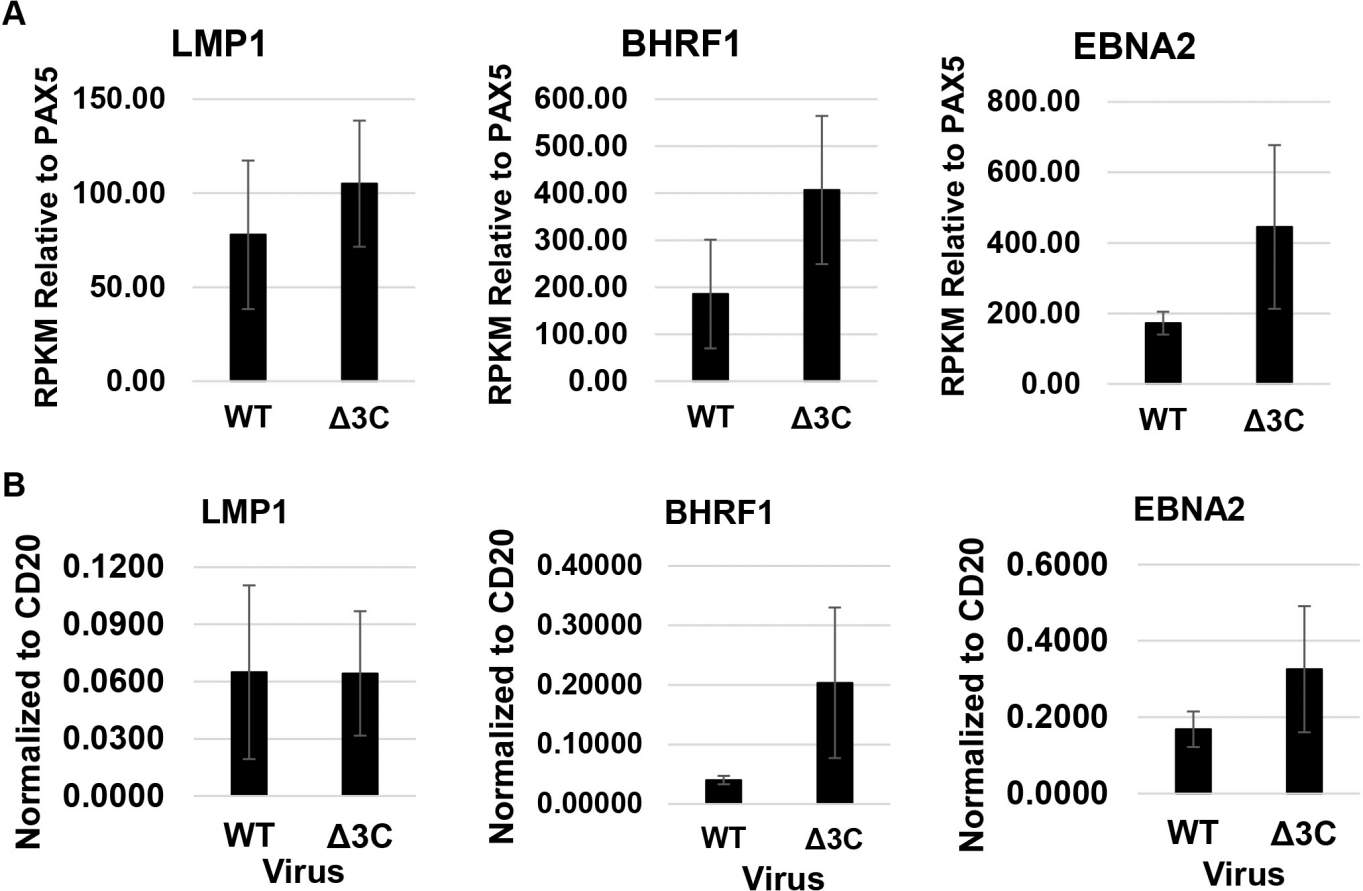
Δ3C virus-induced lymphomas may have increased expression of EBV BHRF1 and EBNA2 transcripts compared to WT virus-induced lymphomas. (A) The average RPKM values of LMP1, EBNA2, and BHRF1, normalized to the PAX5 (B-cell specific) RPKM value of the same tumor, are shown, along with standard error. (B) qPCR analysis of cDNA generated from tumors was performed to quantitate expression of the LMP1, EBNA2, and latent BHRF1 transcripts, respectively. Results were normalized relative to the level of human CD20 (B-cell specific) transcript in each tumor. Standard error is shown.

To examine the pattern of EBV gene expression in two different Δ3C virus-infected, versus two different WT virus-infected, lymphomas, RNA-seq reads were mapped to the EBV genome (Fig 7); transcripts were also mapped to each strand of the viral genome (S6 Fig). Similar levels of lytic gene expression (primarily derived from leftward transcripts) were observed in the presence and absence of EBNA3C (S6 Fig). Interestingly, the two Δ3C-infected tumors appeared to have somewhat increased levels of rightward transcripts mapping to the EBNA2 and BHRF1 genes in comparison to the two WT virus-infected tumors (Figs 7 and S6). In addition, the levels of the leftward LMP1 transcript appeared to be potentially higher in the two Δ3C-infected tumors. Since the RNAseq analysis shown does not normalize the number of viral transcripts to the number of B cells in each specimen, we also compared the number of EBV LMP1, BHRF1 and EBNA2 transcripts to the number of the B-cell specific PAX5 transcript in each tumor sample (Fig 8A), and performed qPCR analysis on cDNA derived from the same tumors (Fig 8B). Although these results suggest that the BHRF1 and EBNA2 transcripts may be higher in the Δ3C-infected tumors, since only two tumors were examined for each tumor type further studies are required to confirm these findings, especially given that the EBNA2 protein levels were similar on immunoblot analysis of tumors (Fig 2B). We did not detect BHRF1 expression by IHC or immunoblot analysis in either tumor type (S7 Fig). It is possible that the increased BHRF1 transcripts in the Δ3C-infected tumors are due to increased levels of the BHRF1 non-coding miRNAs, which are known to promote tumor growth through multiple mechanism(s) [67,68].

### Δ3C virus-infected tumors have reduced expression of E2F target genes in comparison to WT virus-infected tumors

**Fig 9:**
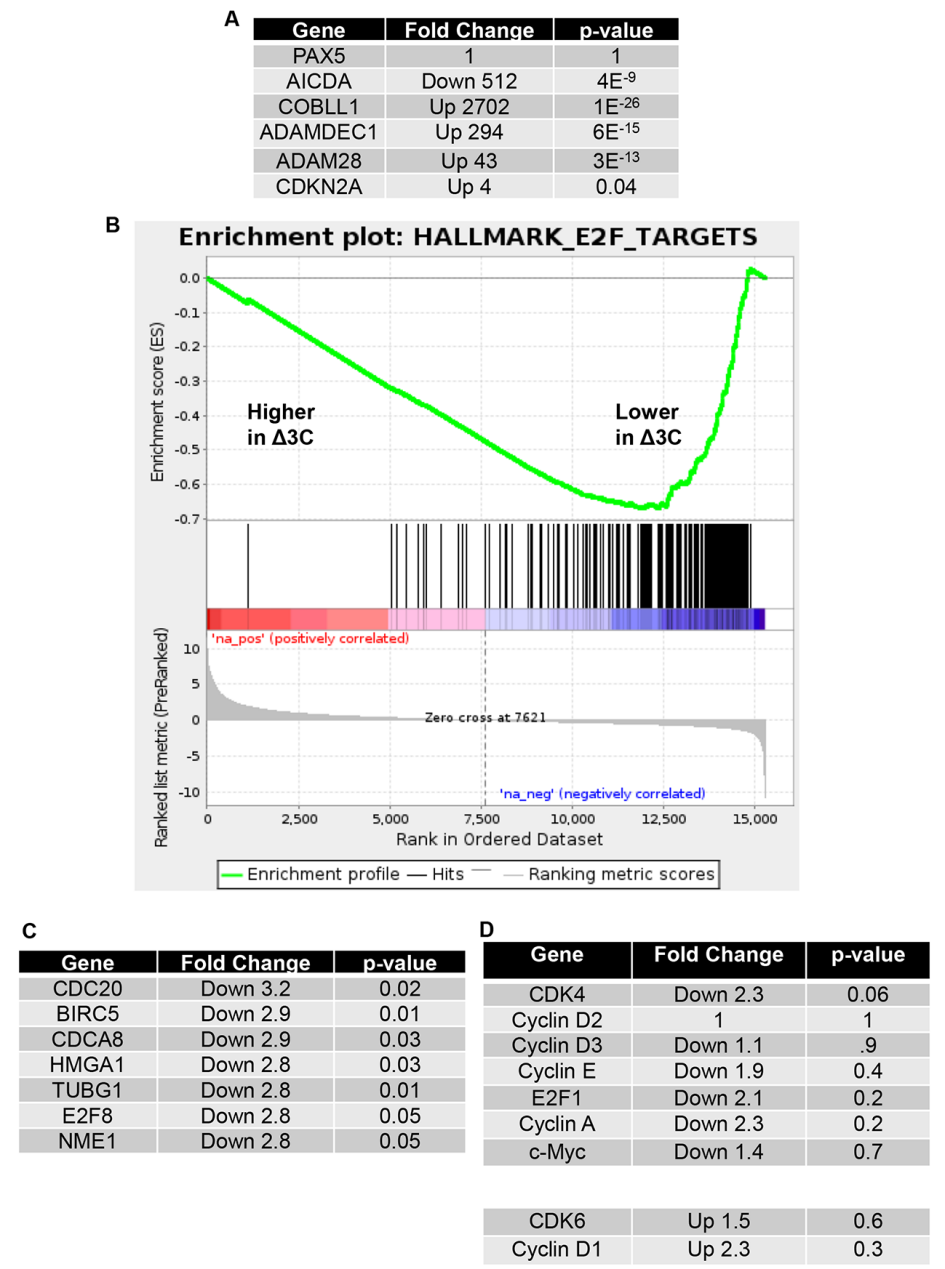
Δ3C virus-induced lymphomas have decreased expression of E2F target genes. RNA was isolated from tumors infected with the WT- or Δ3C virus-induced lymphomas, and RNA-seq performed. Mouse cell transcripts were removed from further analysis, and the levels of human genes in each tumor type was compared as described in the methods. (A). The relative level of cellular gene transcripts in EBNA3C-infected tumors, versus WT virus-infected tumors is shown for PAX5 (a B cell-specific gene), and known EBNA3C target genes. The fold-change in cellular gene expression in EBNA3C-infected tumors versus WT infected tumors is indicated, as well as the p-value for each difference. (B). A gene set enrichment analysis (GSEA) plot for the “Hallmark_E2F_Targets” gene set is shown in Δ3C virus-induced lymphomas compared to WT virus-infected lymphomas. (C). The most downregulated genes in the “Hallmark_E2F_Targets” gene set are shown, along with the fold-change in Δ3C virus–infected versus WT virus-infected cells, and the associated p-value. (D). The relative expression levels of genes encoding proteins important for activation of cell cycle progression are compared in Δ3C virus–infected versus WT virus-infected tumors, and the p-value for differences indicated.

RNA-seq results were also used to compare human cellular gene expression in Δ3C virus-infected versus WT virus-infected tumors. Two tumors of each type had sufficient RNA-seq quality to perform further analysis. RNA-seq results were derived using total tumor tissue, and thus represent a mixture of genes expressed in EBV-infected B cells, infiltrating T cells, and mouse cells (removed by bioinformatic analysis). Since differences in the number of B cells present in each tumor sample, and the presence of infiltrating T cells, could potentially obscure differences in cellular gene expression specific to the B-cell population, we initially compared the expression of a B-cell specific gene, PAX5, and the expression of several different well-known EBNA3C target genes in the Δ3C virus-infected versus WT virus-infected tumors. As shown in Fig 9A, Δ3C virus-infected and WT virus-infected tumors expressed very similar levels of PAX5, suggesting a similar number of B cells in each tumor sample. In contrast, expression of the AICDA gene (activated by EBNA3C *in vitro)* was decreased over 500-fold in Δ3C virus-infected (versus WT virus-infected) lymphomas, and expression of four different cellular genes down-regulated by EBNA3C *in vitro* (CDKN2A, COBLL1, ADAMDEC1, and ADAM28 [26,69]) was significantly increased. These results confirm that RNA-seq analysis of the total tumor cell population detects altered regulation of known EBNA3C target genes in the Δ3C-infected tumors.

To examine the consequence of EBNA3C loss on cellular gene expression more globally, we performed gene set enrichment analysis (GSEA) to identify specific pathways altered in the Δ3C virus-infected (versus WT virus-infected) lymphomas. One of the most significantly down-regulated pathways in the Δ3C-induced tumors was “Hallmark E2F Targets” (Fig 9B and 9C). This result is consistent with increased expression of p16 in Δ3C virus-infected lymphomas (Fig 2C), given that p16 inhibits E2F1 expression by preventing phosphorylation/inactivation of pRb by the cyclin D/CDK4/6 complex [29,33]. E2F1 activates cell cycle progression, and thus decreased expression of E2F target genes is also consistent with our finding that Δ3C virus-infected lymphomas occur less frequently, and at later time points, in comparison to the WT virus-infected lymphomas (Fig 1).

Since Δ3C virus-infected lymphomas continue to proliferate (albeit more slowly) in the presence of high-level p16, we also compared expression of specific cellular genes involved in activating cell cycle progression in the Δ3C-induced versus WT-induced tumors, including cyclin E, CDK4, CDK6, cyclins D1, D2, and D3, c-Myc, E2F1, and cyclin A (Fig 9D). The expression of these genes was not significantly increased or decreased in the Δ3C-induced tumors compared to the WT-induced tumors, although in most cases (except for the cyclin D1 and CDK6 genes) expression was less in the EBNA3C-deleted tumors. The continued transcription of genes required for cell cycle progression, along with c-Myc and cyclin E protein expression (Fig 2C), in the Δ3C virus-infected lymphomas likely allows these tumors to partially escape the effect of high p16 levels.

### Δ3C-induced tumors have an increased type I Interferon response

**Fig 10:**
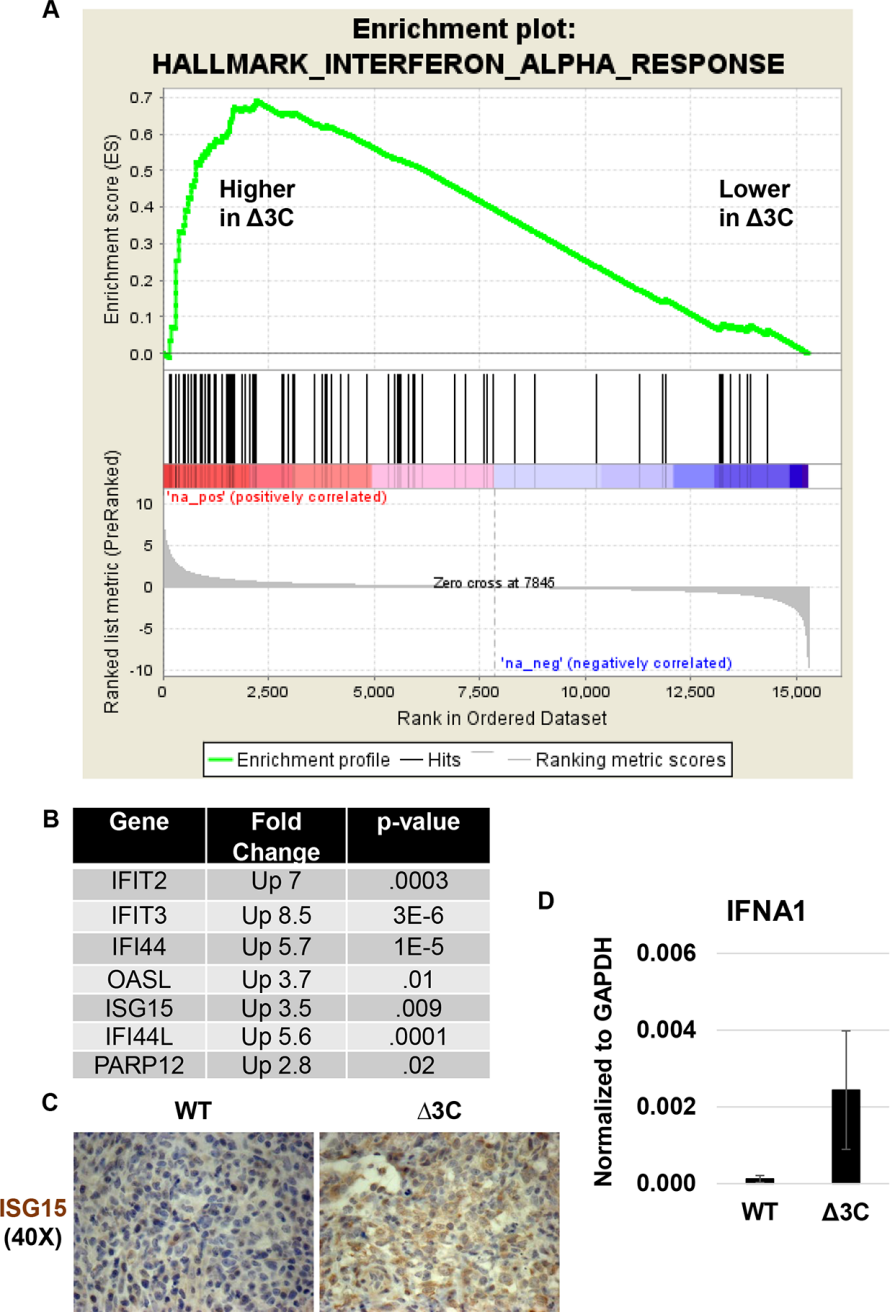
Δ3C virus-induced lymphomas have a gene expression signature suggestive increased type 1 Interferon signaling. (A). GSEA enrichment plot for the “Hallmark_Interferon_Alpha_Respsonse” gene set is shown. (B). Examples of genes in the “Hallmark_Interferon_Alpha_Respsonse” gene set that are of highly upregulated in the Δ3C virus–infected lymphomas relative to the WT virus-infected lymphomas are shown, and p-values are indicated. (C). IHC analysis using antibody targeting ISG15 is shown in tumors infected with the Δ3C virus or WT virus as indicated. (WT: SK1331 and Δ3C: SK1340) (D). qPCR analysis of cDNA generated from tumors was performed to quantitate expression of the human IFNA1 gene (using human specific primers) in WT virus- and Δ3C virus-induced lymphomas. Results were normalized to the level of GAPDH transcript. Standard error is shown.

GSEA analysis also revealed an increase in the “Hallmark Interferon Alpha response pathway” in Δ3C virus-infected tumors (Fig 10A). Examples of alpha interferon-stimulated genes differentially regulated in the Δ3C virus-infected versus WT virus-infected tumors (including IFI44, OASL, ISG15, IFIT2, IFIT3) are shown in Fig 10B. Increased ISG15 protein expression in Δ3C-induced tumors was confirmed using IHC staining (Fig 10C). qPCR analysis of IFNα and IFNβ transcripts in cDNA derived from Δ3C virus-infected versus WT virus-infected tumors revealed increased expression of IFNα (but not IFNβ) in the Δ3C virus-infected tumors (Fig 10D). Since EBV infection of B cells *in vitro* induces a type 1 interferon response [70,71], and type I interferon inhibits EBV-induced transformation of B cells *in vitro* [72], these data suggest that EBNA3C may help to repress this response in EBV-infected lymphomas *in vivo*. Additional studies are required to identify the mechanism(s) underlying this effect and to determine if the source of the interferon signal is primarily B cell- and/or T cell-derived in our model.

### Δ3C-induced tumors have increased expression of T-cell genes and T-cell chemokines

**Fig 11:**
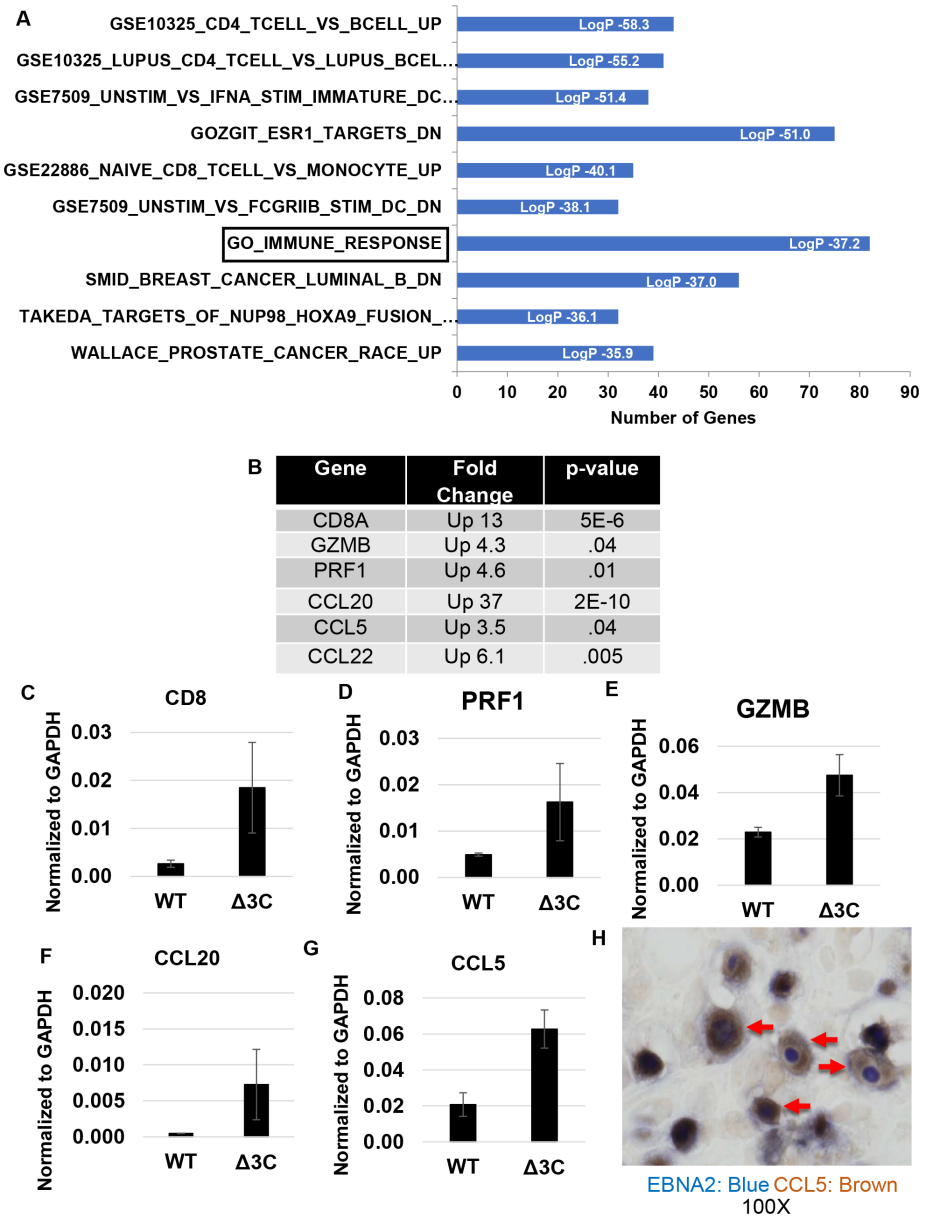
Δ3C virus-induced lymphomas have a signature suggestive of increased T-cell infiltration, and have increased expression of T-cell chemokine genes. (A). Molecular signature pathway analysis of RNA-seq results obtained from Δ3C virus-induced lymphomas versus WT virus-induced lymphomas is shown. (B). Examples of genes included in the “GO_Immune_Response” gene set (boxed in Fig 10A) that are of highly upregulated in the Δ3C virus–infected lymphomas relative to the WT virus-infected lymphomas are shown, and p-values are indicated. (C-G). qPCR was performed using cDNA isolated from Δ3C virus-induced lymphomas versus WT virus-infected lymphomas, using human specific primers to amplify genes shown in Fig 10B. Results were normalized to the level of GAPDH transcript. Standard error is shown. (H). IHC analysis using antibodies against EBNA2 (purple) and CCL5 (brown) in Δ3C virus-induced lymphomas was performed (Δ3C: SK1348). Examples of co-staining cells are indicated with arrows.

Molecular signature analysis of the RNA-seq data also showed a marked increase in pathways associated with T-cell activity in the Δ3C-induced tumors (Fig 11A), consistent with our histologic findings. Differentially regulated genes in one of the molecular signatures, the “GO_Immune_Response gene list” included genes expressed in cytotoxic T cells, including CD8A, perforin1, and granzyme B, as well as T-cell chemokines including CCL5 (RANTES), CCL20, and CCL22 (Fig 11B). The increase in CD8A expression is consistent with our finding that Δ3C-induced tumors have a greater number of infiltrating T cells (Fig 4). We confirmed increased expression of the CD8A, perforin1, granzyme B, CCL5, and CCL20 genes in the Δ3C-induced tumors by performing qPCR analysis on cDNA isolated from tumor tissues (Fig 11C–11G). In addition, we performed IHC co-staining for CCL5 and EBNA2, and detected cells expressing both CCL5 and EBNA2 in Δ3C-induced tumors (Fig 11H). These results suggest that EBNA3C inhibits T-cell responses to EBV-infected B cells by blocking expression of chemokines that attract T cells.

## Discussion

EBNA3C is expressed in human B-cell lymphomas that have type III latency, and in a subset of human Burkitt lymphomas [6,73]. The EBV EBNA3C protein plays an essential role in promoting EBV transformation of B cells into LCLs *in vitro*, and this effect is largely due to the ability of EBNA3C (in collaboration with EBNA3A) to inhibit expression of the CDKN2A tumor suppressor locus. Nevertheless, most EBV-infected lymphomas in humans have restrictive forms of viral latency that do not express EBNA3C [74], indicating that EBV-infected lymphoma cells *in vivo* can sometimes proliferate even without EBNA3C. Furthermore, although EBNA3C regulates numerous different cellular genes in addition to CDKN2A, whether these other EBNA3C-regulated cellular genes have important *in vivo* functions remains unclear.

Here we have used a cord blood-humanized mouse model to examine the phenotype of an EBNA3C-deleted EBV mutant *in vivo* in the presence of human T cells. We find that EBNA3C is not essential for the ability of EBV to cause lymphomas in this model, although mice infected with this mutant have fewer tumors compared to mice infected with wild-type virus, and tumors occur at later time points. Furthermore, we demonstrate that the monoclonal B-cell lymphomas induced by the EBNA3C-deleted mutant occur despite high-level p16 expression and reduced activation of E2F-responsive cellular genes. In addition, we find that loss of EBNA3C expression in EBV-induced lymphomas *in vivo* is associated with enhanced expression of T-cell chemokines, increased T-cell infiltration, and increased type I interferon signaling. These results suggest that EBNA3C is not only critical for reducing p16 expression, but may also affect the T-cell response and type I interferon expression in EBV-infected lymphomas.

Our results confirm that EBNA3C inhibits p16 expression in EBV-infected B cells and show that loss of EBNA3C significantly attenuates the ability of EBV to form lymphomas in cord blood-humanized mice. Nevertheless, the finding that a subset of mice infected with the EBNA3C-deleted virus eventually develop aggressive lymphomas suggests that these tumors have developed mechanism(s) to at least partially thwart the growth inhibitory effect of p16. Although inhibition of p16 transcription via promoter DNA methylation is a relatively common mechanism used by human tumors to prevent p16 accumulation [75], we found that Δ3C virus-infected lymphomas continued to express high-level p16, and thus must have developed other mechanisms to circumvent its growth inhibitory effect. However, this bypass of p16 function was not complete, since a major GSEA signature in Δ3C virus-infected tumors was reduced expression of “Hallmark E2F genes”. This is an expected downstream signature of enhanced p16 expression, since p16 inhibits the ability of cyclin D-CDK4/6 complexes to phosphorylate and inactivate pRb, and pRb inhibits E2F1 transcription.

Although the precise mechanism(s) that allow Δ3C virus-infected lymphomas to proliferate despite high-level p16 expression are not yet fully understood, our results here suggest several (not mutually exclusive) possibilities. Cyclin E/CDK2 complexes (which are not inhibited by p16), like cyclin D-CDK4/6 complexes, can phosphorylate and inactivate pRb, and both human melanomas and DLBCLs expressing high-level p16 have been reported to escape p16 repression by activating cyclin E expression [56–58]. High level c-Myc can likewise allow tumors to escape p16 effects [58]. Our RNA-seq results suggest that cyclin E and c-Myc expression is not significantly lower in Δ3C virus-infected lymphomas versus WT virus-infected lymphomas, and our IHC results showed that both cyclin E and c-Myc are expressed at the protein level in Δ3C virus-infected lymphomas (Fig 2D). Thus, continued c-Myc and/or cyclin E expression likely plays a key role in allowing the Δ3C virus-infected lymphomas to proliferate. EBNA2 activates c-Myc in EBV-infected B cells [9], and we found that Δ3C virus-infected lymphoma cells express at least as much EBNA2 as the wild-type virus-infected lymphomas at both the RNA (Figs 7 and 8) and protein (Fig 2A and 2B) levels. Thus, EBNA2 might promote growth of Δ3C virus-infected lymphomas, even in the presence of high-level p16, by inducing c-Myc (and downstream cyclin E) expression.

Since high-level c-Myc expression (as observed in both the wild-type virus-infected, and Δ3C virus-infected, lymphomas) promotes apoptosis, our findings here also indicate that Δ3C virus-infected lymphomas have developed mechanism(s) to escape c-Myc-induced apoptosis in the absence of EBNA3C. EBNA3C inhibits expression of BIM, a major tumor suppressor for c-Myc-induced B-cell lymphomas, in Burkitt lymphoma cell lines *in vitro* [42]. BIM promotes apoptosis by sequestering anti-apoptosis proteins in the BCL2 family. Although we found that BIM level was significantly decreased in the WT virus-infected versus Δ3C virus-infected lymphomas, BIM was not expressed in most EBV-infected B cells in the Δ3C virus-infected lymphomas, despite the high-level c-Myc expression. Cellular BIM level is controlled through multiple different pathways, including phosphorylation by ERK kinases that promote Bim degradation [76,77], and both LMP1 and LMP2A can activate ERKs [78,79]. LMP1 also activates the NF-kB pathway, which protects B cells from apoptosis by inducing expression of the anti-apoptotic proteins, BCL2 and IRF4. We found that BCL2 and IRF4 are highly expressed in both Δ3C virus-infected, and WT virus-infected lymphomas, even though EBNA3C stabilizes IRF4 protein *in vitro* [43]. These results suggest that the anti-apoptotic functions of other EBV proteins expressed in B cells with type III latency (including LMP1, LMP2A and EBNA3A [80]) at least partially substitute for the anti-apoptotic effect of EBNA3C. In addition, increased transcription of the EBV BCL2 homologue, BHRF1, in Δ3C virus-infected lymphomas (as suggested in Figs 7 and 8) could potentially enhance expression of this latent viral protein may also compensate for loss of EBNA3C expression by inhibiting apoptosis [81]. However, we have been unable to confirm that Δ3C virus-infected lymphomas have increased BHRF1 protein expression by either IHC analysis (perhaps due to inefficient detection with currently available antibodies using IHC staining) or immunoblot analysis (S7 Fig).

In this study, we also demonstrated that EBV-induced tumors in the cord blood-humanized mouse model (both wild-type EBV- and Δ3C virus-induced) are dominated by expansions of a limited number of individual B cells, rather than consisting of highly heterogeneous expansions of a broad selection of different EBV-infected B cells. This finding strongly suggests that the Δ3C virus is driving development of these malignant lymphomas and is not simply persisting as a “passenger virus” in a non-malignant B-cell inflammatory infiltrate. The absence of B-cell lymphomas in NSG mice injected with mock-infected cord blood also confirms that EBV infection is essential for these lymphomas. Interestingly, although the frequency of IGHV2-5 usage in IGH genes expressed in normal cord blood is less than 1% [66], IGHV2-5 usage was observed in the dominant IGH clones in 3 of the 6 tumors examined in this study (Fig 5). IGHV1-69-containing IGH transcripts (obtained from two separate donors) were also over-represented in the EBV-induced tumors in this study relative to the frequency of such transcripts in normal cord blood. These results suggest that expression of specific IGH variable regions may cooperate with EBV infection to induce lymphomas in the cord blood-humanized mouse model, particularly since specific variable IGH gene usage (and specific CDR3 sequences) support the growth of human DLBCLs and CLL tumors by enhancing BCR signaling [82–85]. However, since the antigens recognized by the tumor derived-antibodies in our model are not yet identified, we cannot exclude the possibility that B cells expressing these antibodies are selected for in EBV-infected cord blood-humanized mice because they recognize viral and/or cellular (including mouse) antigens that stimulate their proliferation.

EBNA3C has been reported to increase AID expression in EBV-infected B cells *in vitro* [38], and we found that loss of EBNA3C expression strongly decreased AID expression in EBV-induced lymphomas in the cord blood-humanized model. Although AID is required for IG class-switching and BCR somatic hyper-mutation, we did not find any differences in the amount of class switching or somatic hyper-mutation of antibodies in wild-type virus-infected versus Δ3C virus-infected lymphomas. This lack of difference likely reflects our finding that even the wild-type EBV-infected B cells do not undergo efficient IG class switching or somatic hyper-mutation in the cord blood-humanized mouse model. RNA-seq analysis showed that almost all IG transcripts in both wild-type EBV- and Δ3C virus-infected lymphoma cells were of the IGM rather than IGG types, and our sequencing of the CDR3 motifs of antibodies expressed in lymphomas revealed few if any mutations. The lack of class switching and somatic hyper-mutation in EBV-infected cells may be due to inefficient germinal center-like B cell/T cell interactions in the cord blood-humanized mouse model. Nevertheless, since EBNA3C has been shown to induce somatic hyper-mutation of LCLs *in vitro* even in the absence of any T cell help [38], the previously described ability of EBNA2 to inhibit AID expression and class switching [86,87] may also explain this result. EBNA2-expressing tonsillar cells in patients with infectious mononucleosis do not undergo somatic hyper-mutation [87], consistent with our results here. Thus, the relative level of EBNA2 versus EBNA3C protein expression in EBV-infected B cells may determine whether class switching and somatic hyper-mutation occurs.

In contrast to the restricted B-cell population in tumors, we found that all tumors contained a variety of TCRs, with no single CDR3 identified in multiple tumors. Nevertheless, we did find that certain TRBV genes were used more frequently in some tumors relative to their use in the normal adult peripheral blood cell population. The antigens recognized by the T cell CDR3 motifs in our model are not yet identified and the TCR sequences we observed do not match previously identified “public” TRBV CDR3 motifs reported to recognize EBV antigens. Further studies will be required to determine if the TRBV CDR3 motifs reported here are unique to specific cord blood donors, and to identify the viral and/or cellular antigens recognized by these T cells.

We have also discovered some other, previously unsuspected, potential functions of EBNA3C using the cord blood-humanized mouse model that are difficult to study *in vitro*. The RNA-seq data revealed that Δ3C-induced tumors have a greatly increased type I interferon response compared to the wild-type virus-infected lymphomas, and this effect was confirmed by both qPCR analysis of tumor cDNA, and by ISG15 IHC staining. We also found that IFNα is expressed at higher levels in the Δ3C-induced tumors. Since we used human-specific primers to quantify interferon alpha expression in tumors (Fig 10D), the source of increased interferon alpha must be a human cell. However, technical issues have limited our ability to specifically isolate enough viable B cells and/or T cells from lymphomas to determine if the enhanced interferon response in Δ3C-induced lymphomas is primarily due to interferon produced in B cells, T cells or other cell types. Single-cell RNAseq analysis of lymphomas in this humanized mouse model (in which both human B cells are T cells are intermixed) may help to define the site(s) of interferon production in the future.

The mechanism(s) by which loss of EBNA3C expression leads to an increased interferon response in EBV-infected lymphomas in the cord blood-humanized mouse model is not yet determined. EBER expression in EBV- infected B cells induces strong type I interferon signaling [70,71]. In addition, the ability of EBV infection in B cells to globally induce LTR expression of endogenous retroviruses [88] (a potent stimulator of type I interferon signaling [89,90]), is another potential mechanism by which EBV may induce this pathway. Although we favor a model whereby EBNA3C primarily attenuates the type I interferon response in EBV-infected B cells, the known ability of EBERs to enter uninfected cells via exosomes [91] may also play a role. Although alpha interferon can also be derived from plasmacytoid dendritic cells [92], we have found that EBV-induced lymphomas in cord blood-humanized mice contain relatively few human plasmacytoid dendritic cells. Since interferon alpha inhibits the ability of EBV to transform B cells *in vitro* [72], and blocks its ability to lytically reactivate [93], the ability of EBNA3C to decrease interferon alpha production may enhance both latent and lytic infection. In addition, decreased interferon production in EBV-infected B cells likely benefits the virus *in vivo* by decreasing inflammation and T-cell responses.

Loss of EBNA3C expression also resulted in an increased number of tumor-infiltrating T cells as determined by IHC staining, and RNA-seq data showed increased expression of genes associated with cytotoxic T cells (including CD8, perforin, and granzyme B). RNA-seq analysis also revealed that several different T-cell chemokines (including CCL5, CCL20, and CCL22) are over-expressed in the Δ3C virus-infected, versus WT virus-infected, lymphomas, a finding confirmed by qPCR analysis of tumor cell-derived cDNA. In addition, CCL5 (RANTES) was shown to be expressed at the protein level in Δ3C virus-infected B cells by IHC. These results suggest that EBNA3C inhibits expression of CCL5 in EBV-infected B cells in the cord blood-humanized mouse model. In contrast, we did not find that several different T-cell chemokines previously shown to be inhibited by EBNA3C in an EBV-negative Burkitt cell line *in vitro* (CCL3, CCL4, CXCL10 and CXCL11) [49] were affected by EBNA3C loss in the CBH model, suggesting that the precise effects of EBNA3C may be context-specific. Together, the previous *in vitro* studies, and our results here, suggest that the increased expression of chemokines that attract T cells in Δ3C virus-infected B cells may be a major mechanism leading to increased T-cell infiltration of Δ3C virus-infected lymphomas. However, since the Δ3C virus-infected lymphomas occur at later time points (60–90 days) compared to WT virus-induced lymphomas (30–35 days), we cannot exclude the possibility that the increased time required for lymphomagenesis allows for improved anti-EBV T cell responses, and thus increased T-cell infiltration into lymphomas.

Finally, we suspect that the *in vivo* tumor microenvironment in cord blood-humanized mice may also play a critical role in promoting the development of Δ3C virus-induced tumors. We previously demonstrated that CD40L-expressing CD4 T cells can substitute for LMP1 expression in the cord blood-humanized mouse model [51]. Future studies may identify additional growth factors provided by the *in vivo* tumor microenvironment that allow EBV-infected B cells with stricter forms of viral latency (where the EBV-encoded oncoproteins such as EBNA2, LMP1 and EBNA3C are not expressed) to proliferate in less immunogenic forms of EBV infection.

## Materials and methods

### Ethics statement

All animal work experiments were approved by the University of Wisconsin-Madison Institutional Animal Care and Use Committee (IACUC) and conducted in accordance with the NIH Guide for the care and use of laboratory animals (protocol numbers M005197 and M005214). We anesthetized mice using isoflurane and euthanized animals by performing cervical dislocations on anesthetized mice [53].

### EBV viruses and construction of mutant viruses

All experiments performed in this study were in the context of the p2089 (B95.8 EBV) BACmid. p2089, which expresses green fluorescent protein (GFP) and a hygromycin resistance gene has been described previously [94]. The ΔEBNA3C BACmid was constructed via the GS1783 *E*. *coli-*based *En Passant* method [95,96] by inserting a single nucleotide substitution changing the second amino acid residue to a stop codon. Details on mutagenesis methodology and subsequent derivation of all EBV-positive HEK293 cell lines are described in [97]. Note that because the BM2710 invasive *E*.*coli* (used to infect HEK293 cells) are resistant to Chloramphenicol, the BACmids were made Kanamycin-resistant pre-transfer to the BM2710 *E*.*coli*. To construct ΔEBNA3C-Revert, all steps were reversed starting with the Kanamycin resistant ΔEBNA3C BACmid. The Chloramphenicol insert was created by amplifying the chloramphenicol gene with the corresponding homology arms from the parental p2089 BACmid. All primers used for construction and confirmation of ΔEBNA3C and the corresponding revertant (ΔEBNA3C-Revert) are provided in **Table 1**.

**Table 1:** Primers for constructing EBNA3C mutants.

Primer Name	Sequence	Purpose
EBNA3C-Stop-Fwd	AGATGAGGTAGAAATTTTGCATATTTTCAGACCCACCATGTAATCATTTGAAGGACAGGGGAGGATGACGACGATAAGTAGGG	ΔEBNA3C
EBNA3C-Stop-Rev	TCGGGTGACTGTCTAGAGTCCCCCTGTCCTTCAAATGATTACATGGTGGGTCTGAAAATATCAACCAATTAACCAATTCTGATTAG	ΔEBNA3C
EBNA3C-Revert-Fwd	AGATGAGGTAGAAATTTTGCATATTTTCAGACCCACCATGGAATCATTTGAAGGACAGGGGAGGATGACGACGATAAGTAGGG	ΔEBNA3C Revertant
EBNA3C-Revert-Rev	TCGGGTGACTGTCTAGAGTCCCCCTGTCCTTCAAATGATTCCATGGTGGGTCTGAAAATATCAACCAATTAACCAATTCTGATTAG	ΔEBNA3C Revertant
EBNA3C-Stp-Chk-Fwd	CACGGAATATACCAGGGAAAGG	PCR and sequence
EBNA3C-Stp-Chk-Rev	CCCACTATCGAGTATCAGGTTTG	PCR and sequence
Cam-Ff-Kan-Fwd	CGGGCGTATTTTTTGAGTTATCGAGATTTTCAGGAGCTAAGGAAGCTAAAATGAGCCATATTCAACGGGAAAC	Swap *Chloramphenicol* with *Kanamycin* in F-factor
Cam-Ff-Kan-Rev	CAGGCGTAGCAACCAGGCGTTTAAGGGCACCAATAACTGCCTTAAAAAAATTAGAAAAACTCATCGAGCATC	Swap *Chloramphenicol* with *Kanamycin* in F-factor
2089-CamR-Fwd	CGGGCGTATTTTTTGAGTTATCG	Swap *Kanamycin* with *Chloramphenicol* in F-factor
2089-CamR-Rev	CAGGCGTAGCAACCAGGCG	Swap *Kanamycin* with *Chloramphenicol* in F-factor

### Cell lines and production of infectious EBV

HEK293 cells latently infected with WT EBV or EBNA3C-mutant virus were maintained in Dulbecco modified Eagle medium (DMEM) supplemented with 10% fetal bovine serum (FBS), and 1% penicillin-streptomycin (pen-strep). and 100 ug of Hygromycin B. Infectious viral particles were produced from 293 cell lines stably infected with the WT or mutant viruses following transfection with EBV BZLF1, BRLF1 and gp110 expression vectors as previously described [53]. The titer of EBV was determined on Raji cells by using the green Raji cell assay as previously described [53].

### Creation of cord blood-humanized *NOD/LtSz-scid/IL2Rγ*^*null*^ mice

Immunodeficient NSG (*NOD/LtSz-scid/IL2Rγ*^*null*^) mice were purchased from Jackson labs (catalogue number: 005557). Commercially available CD34-depleted human cord blood mononuclear cells (AllCells) were infected with 5000 Green Raji Units of wild-type or mutant EBV strains *in vitro* for 1 ½ hours at 37°C, and a minimum of 10 million cells were injected intraperitoneally into 3 to 5-week-old NSG mice which were age-matched [51,53]. Mice were kept for a maximum of 90 days after injection of cord blood, or were euthanized due to tumor symptoms prior to day 90. Studies were performed using three different donors.

### Analysis of EBV infection and tumors

Following euthanasia, multiple different organs (including the lungs, spleen, pancreas, liver, gallbladder, mesenteric fat, and abdominal lymph nodes) were formalin fixed and then examined by using a variety of techniques to determine if animals had persistent EBV infection and/or EBV-positive lymphomas and to assess the viral protein expression pattern. Samples from all EBV-infected animals were examined by H&E staining to determine if tumors were present and to assess the types of tumors in each animal. Tumors from at least 10 different animals infected with the Δ3C or wild-type viruses also underwent IHC staining by using the antibodies listed in **Table 2**, as previously described [53,98]. Coauthor Erik A. Ranheim, a board-certified hematopathologist, performed the pathological analysis of the tumors. In some animals, EBER *in situ* hybridization studies were performed by using the PNA ISH detection kit (DakoCytomation) as previously described [53]. For quantification of IHC results, at least 2 random fields of view were selected per animal, photographed, and then counted by 2 independent observers. The counts of positively staining cells were averaged across observers. For each ratio (i.e., CD3 to CD20), stains were done on adjacent slides, and counts were completed on the same field of view. These analyses were performed on 5 WT tumors, 5 revertant virus tumors, and 10 Δ3C virus tumors (S3 Table), using which tumors from all three donors.

**Table 2:** Antibodies used for immunohistochemistry and immunoblot.

Antibody	Clone	Manufacturer	Dilution
CD20 (mouse)	H1	BD Pharmingen	1:500
CD20 (rabbit)	BV11	Abcam, Inc.	1:100
CD3	Polyclonal	DakoCytomation	1:200
LMP1 (IHC)	CS. 1–4	Abcam, Inc.	1:10
LMP1 (Immunoblot)	CS. 1–4	Abcam, Inc.	1:500
EBNA2	PE2	Abcam, Inc.	1:100
EBNA1 (IHC)	1EBI14	Santa Cruz Biotechnology	1:100
EBNA1 (Immunoblot)	O211	Santa Cruz Biotechnology	1:500
EBNA3C	Polyclonal	Exalpha	1:500
BHRF1 (EA-R-p17)	5B11	Millipore	1:500
BIM	C34C5	Cell Signaling Technology	1:100
IRF4	MUM1p	Santa Cruz Biotechnology, Inc.	1:50
P16INK4a	JC8	NeoMarkers	1:100
Cyclin E	HE12	Santa Cruz Biotechnology, Inc.	1:100
c-Myc	Y69	Abcam, Inc.	1:50
BCL2	C-2	Santa Cruz Biotechnology, Inc.	1:100
ISG15	Polyclonal	GeneTex	1:400
CCL5 (RANTES)	Polyclonal	Abcam, Inc.	1:250
Actin	AC-15	Sigma	1:5000

Antibodies used for to detect cellular and viral proteins by IHC and Immunoblot are indicated.

### Immunoblot analysis of tumor protein extracts

Frozen tumor samples were homogenized using Cellcrusher tissue pulverizer system (Cellcrusher) as per the manufacturer’s instructions. Immunoblotting was performed as described previously [99]. Briefly, tumor tissue was lysed in SUMO buffer plus protease inhibitors and then sonicated and centrifuged. Equivalent amounts of protein were separated in sodium dodecyl sulfate-10% or 15% polyacrylamide gel electrophoresis (SDS-PAGE) gels and transferred to nitrocellulose membranes. Membranes were blocked in 5% milk and then incubated with the appropriate primary antibodies diluted in 5% milk in 1X PBS and 0.1% Tween 20 (PBS-T). The primary antibodies used are described in **Table 2**. After being washed, the membranes were incubated with the appropriate horseradish peroxidase-conjugated secondary antibodies (Pierce, Waltham, MA) in 5% milk–1X PBS-T for 1 h at room temperature and then washed again. Bound antibodies were visualized by use of enhanced chemiluminescent reagent (Pierce) according to the manufacturer’s instructions.

### RNA-seq analysis of tumor tissue

RNA samples were harvested from frozen primary tumor tissue by homogenizing tissue with the Covaris Cryoprep Pulverizer. The pulverized tissue was then lysed in Trizol reagent (Invitrogen). RNA was isolated using phenol/chloroform extraction and total RNA was quantitated using a NanoDrop 2000 Spectrophotometer machine (ThermoFisher). RNA-seq libraries were prepared using the TruSeq Stranded mRNA Prep kit (illumina). Libraries were sequenced using an illumina HiSeq2500 platform at the University of Wisconsin Biotechnology Center DNA Sequencing Facility.

RNA Seq data analysis was conducted by BioInfoRx (Madison, WI). Briefly, the fastQC program was used to verify raw data quality of the Illumina reads. The sequence data were mapped to human, mouse and virus genomes separately. The hg19 human genome and Ensembl gene annotations (v75), GRCm38 (mm10) mouse genome and Ensembl gene annotations (v82) were used for mapping. The raw sequence reads were mapped to the genome using Subjunc aligner from Subread [100]; then, we took the human and mouse alignment files (bam files) and assigned a read to one of the following categories (in the order listed below): 1. Mapped to both (mapping scores for both human and mouse >20, the difference between human and mouse mapping scores < = 10); 2. Mapped mostly to human (human mapping score>20, mapping to human genome better than to mouse genome (higher mapping score and fewer mismatches)); 3. Mapped mostly to mouse (mouse mapping score>20, mapping to mouse genome better than to human genome (higher score and fewer mismatches)); 4. Mapped to human (human mapping score minus mouse mapping score >10); 5. Mapped to mouse (mouse mapping score minus human mapping score >10); 6. No mapping (both mapping scores are 0); 7. Others. Reads from categories 2 and 4 were combined as human only reads (17–42% of all reads), and reads from categories 3 and 5 were combined as mouse only reads (18–42% of all reads). These mouse only and human only alignment bam files were compared against corresponding gene annotation GFF files, and raw counts for each gene were generated using the featureCounts tool from Subread, with around 30–47% of reads overall assigned to human genes, and around 31–51% of reads overall assigned to mouse genes. The raw counts data were normalized using the TMM normalization method [101] in the program edgeR, and the normalized gene counts were transformed to log2 scale using the voom method from the R Limma package [102], then used for differential expression analysis. Functional interpretation of the differentially expressed genes was conducted based on GO terms, KEGG pathway and GSEA [103] methods.

### EBV genome analysis

EBV transcripts were analyzed by aligning the fastq files to an indexed B95-8 EBV genome using Burrows-Wheeler Aligner (BWA) [104]. SAMtools was used to generate sorted BAM files. A pileup of aligned reads was constructed as a Wig file using a python script. RPKM measurements for EBV genes were derived by normalizing the number of aligned reads to the total number of reads aligned to human or EBV transcripts.

### Quantitative PCR analysis of tumor tissue

cDNA was made from RNA collected from frozen tumor tissues using methods described above. The extracted RNA was then treated with DNase, followed by reverse transcription using random primers and GoScript reverse transcriptase (RT) (Promega). Real-time PCR was performed on the reverse-transcribed cDNA by using iTaq Universal SYBR green mix (Bio-Rad) in a Bio-Rad CFX96 machine. cDNA (1 uL) was used for 40 cycles of 15 s at 95°C and 30 s at 60°C [105]. The CFX Maestro software (Biorad) was used to collect Cq values. Cq values were either normalized to the CD20 (MS4A1) value (EBV genes and B-specific genes) or total GAPDH. Delta Cq values were determined by calculating 2^ (Normalized Cq) which were then plotted. All primers used in qPCR analysis are listed in **Table 3**[105,106].

**Table 3:** Primers for qPCR analysis.

Gene	Forward Primer (5’-3’)	Reverse Primer (5’-3’)
GAPDH	TGCACCACCAACTGCTTAG	GATGCAGGGATGATGTTC
CD20 (MS4A1)	GCTGGCATCGTTGAGAATGAAT	TGCTGACAGGAGAACTATGTTAGAT
LMP1	TGAGTAGGAGGGTGA	CTATTCCTTTGCTCTCATGC
Latent BHRF1	TACGCATTAGAGACCACTTTGAGCC	TTCTCTTGCTGCTAGCTCCA
EBNA2	TACGCATTAGAGACCACTTTGAGCC	AAGCGCGGGTGCTTAGAAGG
IFNA1	TAGACAAATTCTGCACCGAAC	AGATGGAGTCCGCATTCATC
CD8	TGTGAGGGGCTCTCCAACAA	AGGCCCTCTTGAATCTCTGAATTT
Perforin 1	CCCAGTGGACACACAAAGGTT	TCGTTGCGGATGCTACGAG
Granzyme B	CGATGATCTCCCCTGCATCTG	CCTTCCTGAGAAGATGCAACCA
CCL20	GGCGAATCAGAAGCAGCAAGC	CTGCCGTGTGAAGCCCACAATA
CCL22	GCGCGTGGTGAAACACTTCT	CCCTGAAGGTTAGCAACACCA
CCL5	TCAAGGACTCTCCATCCTAGCTC	AACCCAGCAGTCGTCTTTGTC

Forward and reverse primers used for qPCR analyses of various cellular and viral genes are indicated.

### B-cell immunoglobulin and T-cell receptor sequencing

FASTQ files were first processed with Trim Galore! v0.4.4 (Cutadapt v1.14) with default parameters to remove adapter sequences and low quality bases and reads. Following trimming, BCR and TCR clonotypes were predicted using miXCR (v2.1.11) as described in the documentation [107]. MiXCR extendAlignments was used to extend incomplete TCR CDR3 sequences using germline annotations. The amount of somatic hyper-mutation in IGHV sequences was analyzed using IMGT/Vquest software (http://www.imgt.org/IMGT_vquest/vquest).

### Statistical analysis

All bar graphs were constructed in Microsoft Excel and standard error was used for error bars in graphs. Kaplan-Meier analysis and dot plots were constructed in MSTAT statistical software. Fisher Exact, Log-Rank analysis and Wilcoxon rank-sum test was performed using MSTAT statistical software (https://mcardle.wisc.edu/mstat/). A p-value of < .05 was considered significant in all tests used.

### Data accessibility

The RNA-seq data reported in this paper have been deposited in the GEO database and are under the GEO accession number GSE113070.

## Supporting information

S1 Protocol Isolation of DNA from FFPE tissues. (DOCX)

S1 Fig Δ3C-induced lymphomas retain the presence of EBNA3C mutation. DNA was isolated from FFPE slides of lymphoma tissues isolated from animals infected with WT or Δ3C viruses, and PCR amplified using EBNA3C specific primers (Table 1) to obtain the EBNA3C sequence. The Δ3C-induced lymphomas retained the inserted stop codon mutation as shown.
(PDF)

S2 Fig EBNA2 and LMP1 are co-expressed in lymphoma cells in tumors infected with WT or Δ3C viruses. Co-staining was performed on both WT- and Δ3C-indcued lymphomas using antibodies against EBNA2 (blue) and LMP1 (Brown). The majority of LMP1-expressing cells also expressed EBNA2 (indicative of type III latency) in tumor cells infected with either the WT or Δ3C viruses (WT: SK1498 and Δ3C: SK1501). Examples of co-staining cells are indicated with arrows.
(PDF)

S3 Fig The frequency of EBV-infected B cells in tumors is similar in WT- and Δ3C-induced lymphomas. (A) IHC and ISH (EBER) staining was performed on adjacent slides to detect CD20 (B cell marker), EBNA1 (EBV latent protein), and EBERs as indicated (WT: SK1332 and Δ3C: SK1340). (B) Quantification of the ratio of EBER+ cells to CD20+ cells in 9 different tumors from each condition is shown. (C) Quantification of the ratio of EBNA1+ cells to CD20+ cells in 8 different tumors infected with either virus type.
(PDF)

S4 Fig Δ3C-induced lymphomas have an increased number of CD3+ T cells but a similar number of CD20+ B cells in comparison to WT-induced lymphomas. The total number of CD20+ and CD3+ cells per 40X field is shown for 8 tumors infected with each virus type. The Δ3C-induced lymphomas have similar total number of B cells as WT-induced lymphomas but have an increased total number of T cells.
(PDF)

S5 Fig Sequence analysis of BCR CDR3s in EBV-infected lymphomas. The dominant VDJ recombination, CDR3 protein sequence, and number of nucleotide mutations was determined for each tumor as described in the methods. The specific CDR3 sequence for each tumor is shown and compared to the expected germline sequence. Mutations that do not alter protein sequence are labelled silent (green) and mutations that alter protein sequence are labelled non-silent (yellow).
(DOCX)

S6 Fig Strand-specific analysis of EBV transcripts. RNAseq reads that originated from either strand of the EBV genome are shown. The expression of lytic genes (which are largely leftward) in the Δ3C-induced lymphomas is similar to WT-induced lymphomas (very low in each case).
(PDF)

S7 Fig WT and Δ3C virus infected lymphomas do not express the EBV BHRF1 protein. Protein derived from lymphomas infected with WT or Δ3C viruses was used to perform immunoblots to detect BHRF1 and actin as indicated. Lytically induced (I) or un-induced (U) B95.8 marmoset cells served as positive and negative controls for BHRF1 protein expression.
(PDF)

S1 Table Detailed description of tumors infected with WT versus Δ3C viruses. Characteristics of the tumors used in this study are shown (including the virus used to infect animals, the time of euthanasia, and the anatomic sites invaded by each of the various tumors).
(DOCX)

S2 Table WT and mutant tumors have similar numbers of EBV-infected B cells. The raw values for the number of EBER, EBNA1, and CD20 positive cells per 40X field view are shown for tumors infected with WT versus Δ3C viruses. ND indicates samples where EBER or EBNA1 positive cells were not quantified.
(DOCX)

S3 Table Tumors included within each figure. The tumor ID numbers included in each figure and Table, and the type of virus infection in each tumor, is shown.
(XLSX)

S4 Table TCRB CDR3 sequences from wild-type (WT) EBV and Δ3C EBV infected lymphomas. CDR3 sequences of TCRB transcripts were deducted from RNA-seq analysis as described in the methods.
(DOCX)

## Note in Proof

While this manuscript was being revised, another group reported that an EBNA3C-deleted virus is able to establish latency in another humanized mouse model [108].
