# Supplementary material for: An EBNA3C-deleted Epstein-Barr virus (EBV) mutant causes B-cell lymphomas with delayed onset in a cord blood-humanized mouse model
Source: PLoS Pathog. 2018 Aug 20;14(8):e1007221. doi: 10.1371/journal.ppat.1007221 (PMC6117096; doi:10.1371/journal.ppat.1007221)
Supplement: S1 Protocol — (DOCX) [file ppat.1007221.s001.docx]

**S1 Protocol**

**Isolation of DNA from FFPE tissues:** DNA was isolated from Formalin-fixed paraffin embedded (FFPE) tissues on slides. We isolated DNA from FFPE tissue using the QIAamp DNA FFPE tissue kit (Qiagen) per the manufacturer’s protocol. DNA concentration was determined using the NanoDrop 2000 Spectrophotometer machine (ThermoFisher). PCR analysis was performed on DNA using primer described in **Table 1** (EBNA3C-Stp-Chk primers) using the following amplification conditions: 95^o^C for 10 min, 95^o^C for 15 sec, 49.3^o^C for 30 sec, 72^o^C for 54 sec, repeat 40 times, 72^o^C for 5 min. The PCR product was isolated and submitted for Sanger sequencing at the UW biotechnology center. Sequences were analyzed using Seq Scanner 2 (Applied Biosciences).
