# Supplementary figures and images for: An EBNA3C-deleted Epstein-Barr virus (EBV) mutant causes B-cell lymphomas with delayed onset in a cord blood-humanized mouse model
Source: PLoS Pathog. 2018 Aug 20;14(8):e1007221. doi: 10.1371/journal.ppat.1007221 (PMC6117096; doi:10.1371/journal.ppat.1007221)

S1 Figure

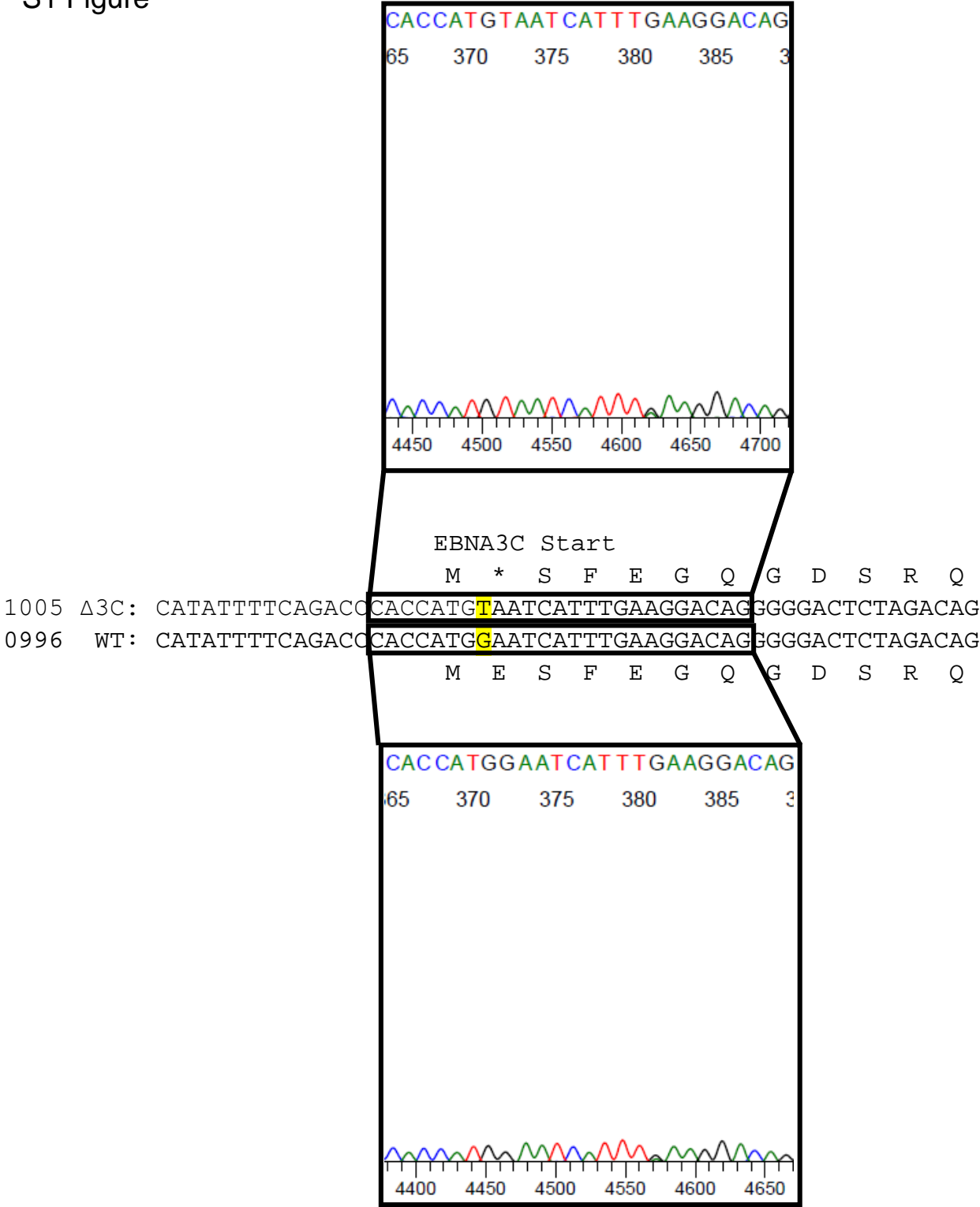

Supplement: S1 Fig — DNA was isolated from FFPE slides of lymphoma tissues isolated from animals infected with WT or Δ3C viruses, and PCR amplified using EBNA3C specific primers (Table 1) to obtain the EBNA3C sequence. The Δ3C-induced lymphomas retained the inserted stop codon mutation as shown. (PDF) [file ppat.1007221.s002.pdf]

S2 Figure

**WT**

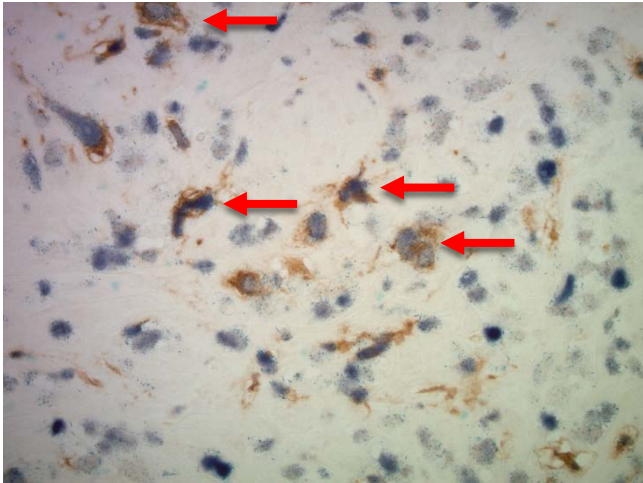

**$\Delta$ 3C**

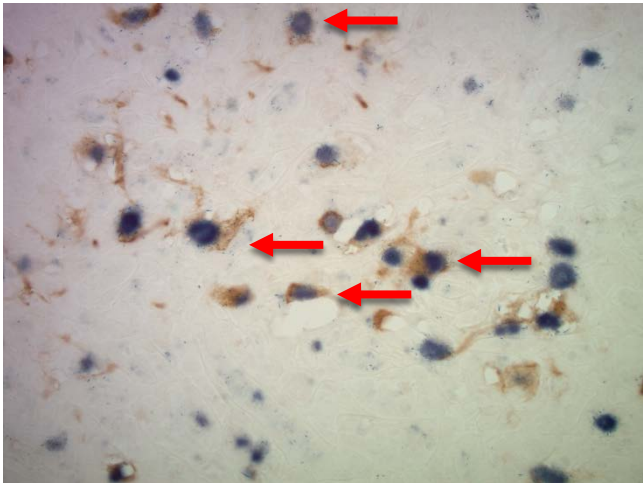

**EBNA2 & LMP1 (40X)**

Supplement: S2 Fig — Co-staining was performed on both WT- and Δ3C-indcued lymphomas using antibodies against EBNA2 (blue) and LMP1 (Brown). The majority of LMP1-expressing cells also expressed EBNA2 (indicative of type III latency) in tumor cells infected with either the WT or Δ3C viruses (WT: SK1498 and Δ3C: SK1501). Examples of co-staining cells are indicated with arrows. (PDF) [file ppat.1007221.s003.pdf]

S3 Figure

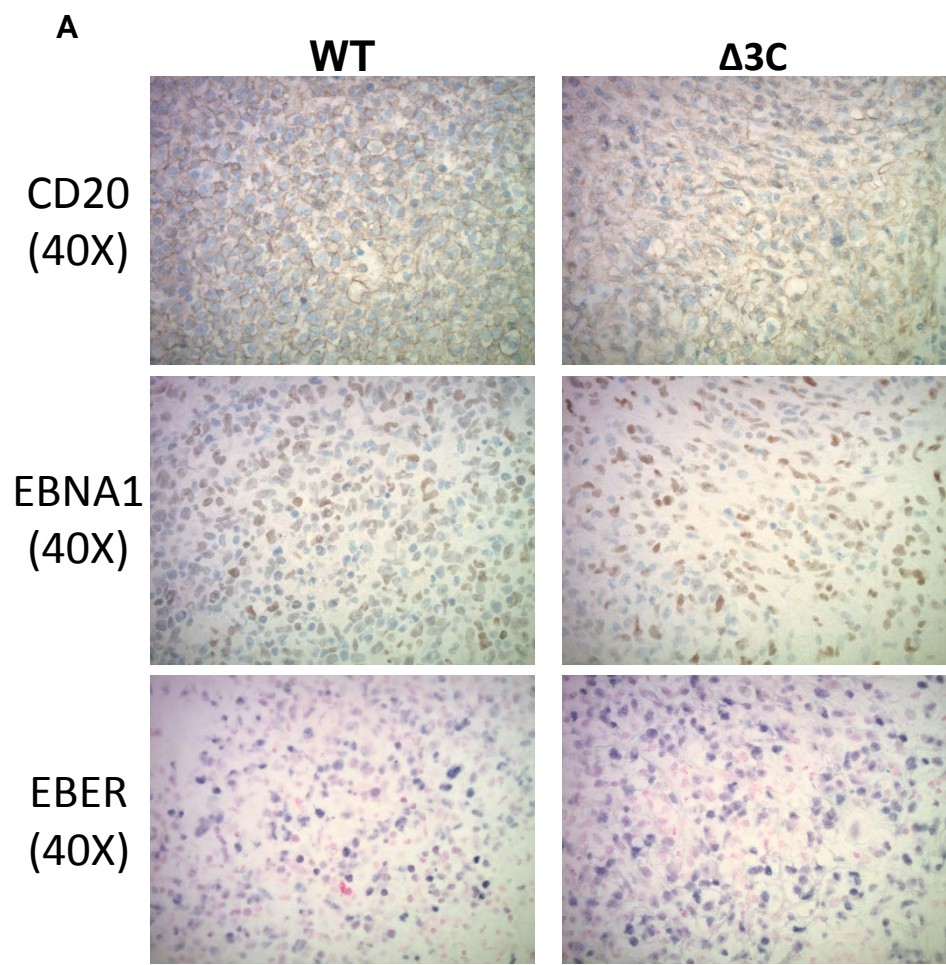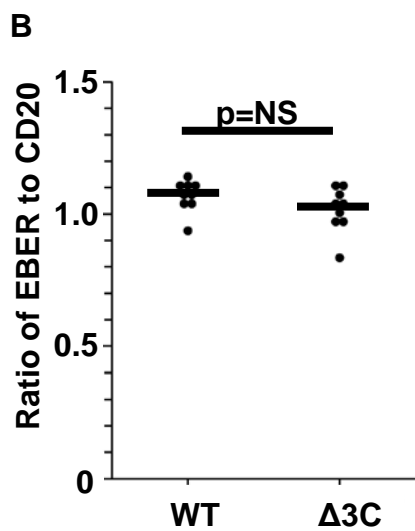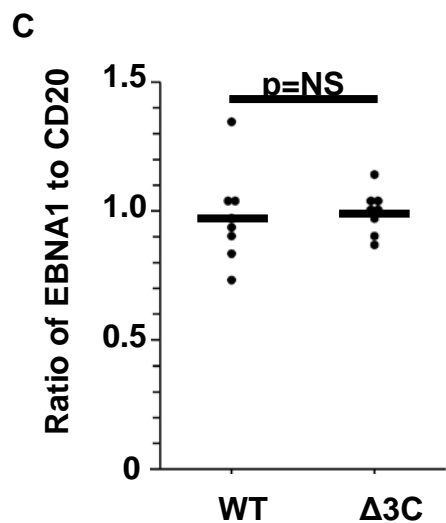

Supplement: S3 Fig — (A) IHC and ISH (EBER) staining was performed on adjacent slides to detect CD20 (B cell marker), EBNA1 (EBV latent protein), and EBERs as indicated (WT: SK1332 and Δ3C: SK1340). (B) Quantification of the ratio of EBER+ cells to CD20+ cells in 9 different tumors from each condition is shown. (C) Quantification of the ratio of EBNA1+ cells to CD20+ cells in 8 different tumors infected with either virus type. (PDF) [file ppat.1007221.s004.pdf]

S4 Figure

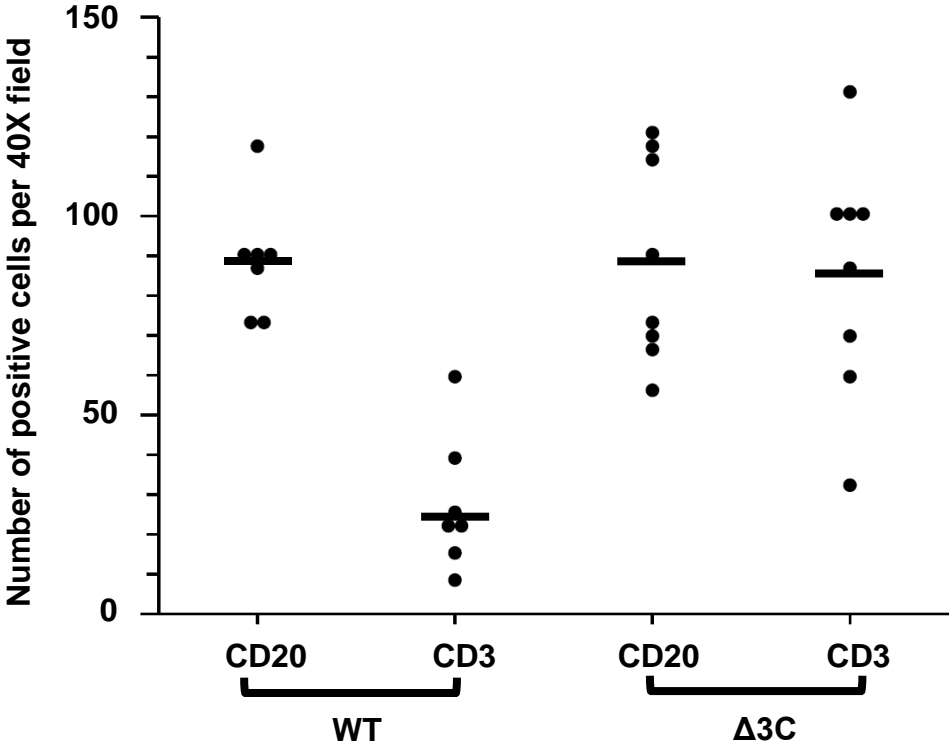

Supplement: S4 Fig — The total number of CD20+ and CD3+ cells per 40X field is shown for 8 tumors infected with each virus type. The Δ3C-induced lymphomas have similar total number of B cells as WT-induced lymphomas but have an increased total number of T cells. (PDF) [file ppat.1007221.s005.pdf]

S6 Figure

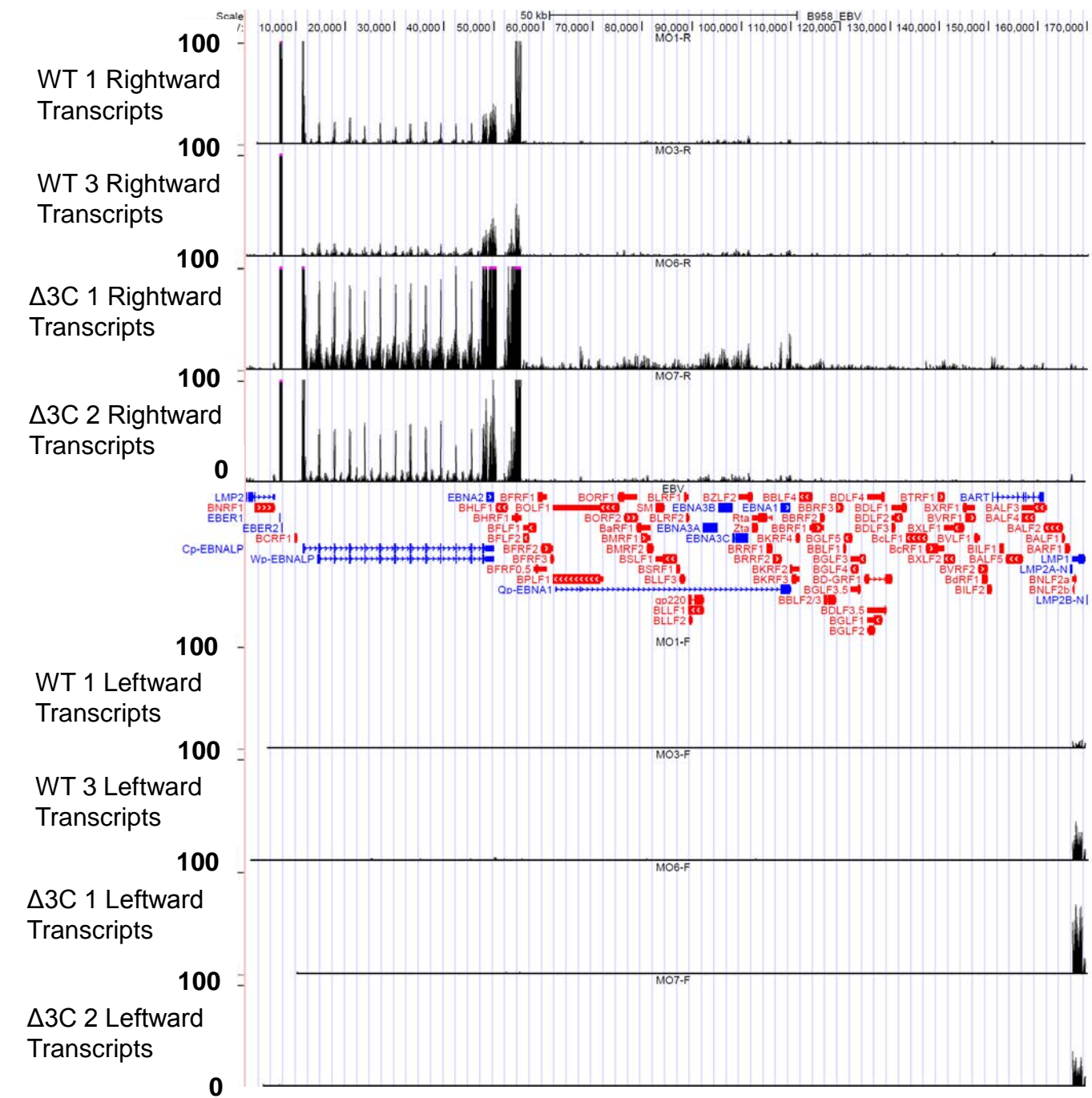

Supplement: S6 Fig — RNAseq reads that originated from either strand of the EBV genome are shown. The expression of lytic genes (which are largely leftward) in the Δ3C-induced lymphomas is similar to WT-induced lymphomas (very low in each case). (PDF) [file ppat.1007221.s007.pdf]

S7 Figure

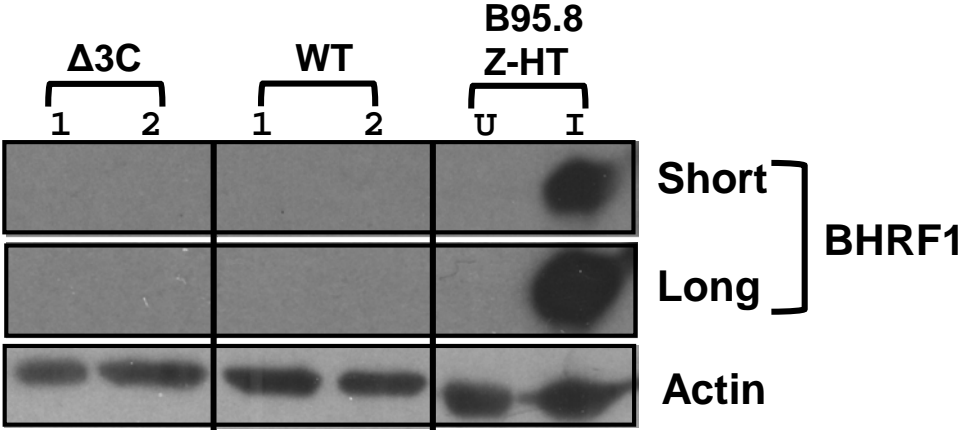

Supplement: S7 Fig — Protein derived from lymphomas infected with WT or Δ3C viruses was used to perform immunoblots to detect BHRF1 and actin as indicated. Lytically induced (I) or un-induced (U) B95.8 marmoset cells served as positive and negative controls for BHRF1 protein expression. (PDF) [file ppat.1007221.s008.pdf]
