# Supplementary material for: An EBNA3C-deleted Epstein-Barr virus (EBV) mutant causes B-cell lymphomas with delayed onset in a cord blood-humanized mouse model
Source: PLoS Pathog. 2018 Aug 20;14(8):e1007221. doi: 10.1371/journal.ppat.1007221 (PMC6117096; doi:10.1371/journal.ppat.1007221)
Supplement: S5 Fig — The dominant VDJ recombination, CDR3 protein sequence, and number of nucleotide mutations was determined for each tumor as described in the methods. The specific CDR3 sequence for each tumor is shown and compared to the expected germline sequence. Mutations that do not alter protein sequence are labelled silent (green) and mutations that alter protein sequence are labelled non-silent (yellow). (DOCX) [file ppat.1007221.s006.docx]

S5 Figure

WT Tumor 1

IGHV2-5 D3-10 J3 Sequence

C A H S Y G E V G S P D A F D I W

Tumor Sequence: TGTGCACACTCATACGGGGAGGTGGGTTCCCCTGATGCTTTTGATATCTGG

Germline Seq: TGTGCACACAGACACGGGGAGGTGGGTTCCCCTGATGCTTTTGATATCTGG

C A H R Y G E V G S P D A F D I W

V2-5 D3-10 J3

2 non-silent mutations (A319>T and G230>C)

1 silent mutation (C322>T)

WT Tumor 2

IGHV2-5 D6-25 J4 Sequence

C A H R P S G Y Y F D Y W

Tumor Sequence: TGTGCACACAGACCGAGCGGGTACTACTTTGACTACTGG

Germline Seq: TGTGCACACAGACCGAGCGGGTACTACTTTGACTACTGG

C A H R P S G Y Y F D Y W

V2-5 D6-25 J4

O mutations

WT Tumor 3

IGHV1-69 D6-13 J6 Sequence

C A R D L A A A G T Y Y Y Y Y G M D V W

Tumor Sequence: TGTGCGAGAGATCTAGCAGCAGCTGGTACTTACTACTACTACTACGGTATGGACGTCTGG

Germline Seq: TGTGCGAGAGATCTAGCAGCAGCTGGTACTTACTACTACTACTACGGTATGGACGTCTGG

C A R D L A A A G T Y Y Y Y Y G M D V W

V1-69 D6-13 J6

0 mutations

Δ3C Tumor 1

IGHV3-73 D1-26 J4 Sequence

C T R L T L V G A T D Y W

Tumor Sequence: TGTACTAGATTGACCCTAGTGGGAGCTACTGACTACTGG

Germline Seq: TGTACTAGACAGACCCTAGTGGGAGCTACTGACTACTGG

C T R L T L V G A T D Y W

V3-73 D1-26 J4

2 silent mutations (C319>T and C320>A)

IGHV1-69 D7-27 J3 Sequence

C A R E L T G D A F D I W

Tumor Sequence: TGTGCGAGAGAGTTAACTGGGGATGCTTTTGATATCTGG

Germline Seq: TGTGCGAGAGAGTTAACTGGGGATGCTTTTGATATCTGG

C A R E L T G D A F D I W

V1-69 D1-26 J3

0 Mutations

Δ3C Tumor 2

IGHV3-23 D7-27 J4 Sequence

C A K A L K A I N W G L D Y w

Tumor Sequence: TGTGCGAAAGCCTTAAAAGCGATTAACTGGGGTCTTGACTACTGG

Germline Seq: TGTGCGAAAGACTTAAAAGCGATTAACTGGGGTCTTGACTACTGG

C A K A L K A I N W G L D Y w

V3-23 D7-27 J4

1 silent mutation (A320>C)

Δ3C Tumor 3

IGHV2-5 D2-21 J4 Sequence

C A H S K E V T A I L F D Y W

Tumor Sequence: TGTGCACACAGCAAGGAGGTGACTGCTATTTTATTTGACTACTGG

Germline Seq: TGTGCACACAGACAGGAGGTGACTGCTATTTTATTTGACTACTGG

C A H R K E V T A I L F D Y W

V2-5 D2-21 J4

1 non-silent mutation (A321>C)

1 silent mutation (C322>A)

Yellow = non-silent mutation

Green = silent mutation
