# Supplementary material for: An EBNA3C-deleted Epstein-Barr virus (EBV) mutant causes B-cell lymphomas with delayed onset in a cord blood-humanized mouse model
Source: PLoS Pathog. 2018 Aug 20;14(8):e1007221. doi: 10.1371/journal.ppat.1007221 (PMC6117096; doi:10.1371/journal.ppat.1007221)
Supplement: S1 Table — Characteristics of the tumors used in this study are shown (including the virus used to infect animals, the time of euthanasia, and the anatomic sites invaded by each of the various tumors). (DOCX) [file ppat.1007221.s009.docx]

**S1 Table: Detailed description of tumors infected with WT versus Δ3C viruses**

| Tumor ID | Virus | Day of Euthanasia | Location of Tumors |
| --- | --- | --- | --- |
| SK999 | WT | 35 | Pancreas, Biliary tract |
| SK1002 | WT | 35 | Pancreas, Biliary tract, Gallbladder |
| SK1189 | WT | 30 | Pancreas, Liver, Mesentery, Small Intestine (wall) |
| SK1191 | WT | 30 | Pancreas, Gallbladder, Liver, Mesentery |
| SK1193 | WT | 30 | Pancreas, Gallbladder, Mesentery |
| SK1331 | Δ3C Revertant | 35 | Mesentery, Diaphragm |
| SK1332 | Δ3C Revertant | 31 | Pancreas, Biliary tract, Mesentery |
| SK1335 | Δ3C Revertant | 35 | Pancreas, Mesentery |
| SK1338 | Δ3C Revertant | 35 | Pancreas, Mesentery, Diaphragm |
| SK1339 | Δ3C Revertant | 35 | Pancreas, Biliary tract |
| SK1003 | Δ3C | 66 | Biliary tract, Gallbladder |
| SK1005 | Δ3C | 66 | Pancreas, Biliary tract, Liver |
| SK1183 | Δ3C | 66 | Pancreas, Biliary tract, Gallbladder |
| SK1192 | Δ3C | 65 | Pancreas, Mesentery |
| SK1194 | Δ3C | 65 | Pancreas, Mesentery |
| SK1337 | Δ3C | 73 | Pancreas, Biliary tract |
| SK1340 | Δ3C | 74 | Pancreas |
| SK1347 | Δ3C | 90 | Pancreas, Mesentery |
| SK1348 | Δ3C | 90 | Pancreas, Biliary tract |
| SK1350 | Δ3C | 90 | Pancreas, Biliary tract, Small Intestine (wall) |
