# Supplementary material for: An EBNA3C-deleted Epstein-Barr virus (EBV) mutant causes B-cell lymphomas with delayed onset in a cord blood-humanized mouse model
Source: PLoS Pathog. 2018 Aug 20;14(8):e1007221. doi: 10.1371/journal.ppat.1007221 (PMC6117096; doi:10.1371/journal.ppat.1007221)
Supplement: S2 Table — The raw values for the number of EBER, EBNA1, and CD20 positive cells per 40X field view are shown for tumors infected with WT versus Δ3C viruses. ND indicates samples where EBER or EBNA1 positive cells were not quantified. (DOCX) [file ppat.1007221.s010.docx]

**S2 Table: WT and mutant tumors have similar numbers of EBV-infected B cells.**

| Animal ID | Average Number per 40X field | | Average Number per 40X field | |
| --- | --- | --- | --- | --- |
| WT | EBER | CD20 | EBNA1 | CD20 |
| 999 | 106 | 96 | 85 | 96 |
| 1002 | 107 | 97 | 95 | 97 |
| 1189 | 71 | 76 | 78 | 76 |
| 1191 | 94 | 82 | 111 | 82 |
| 1331 | 103 | 95 | 97 | 95 |
| 1332 | 47 | 45 | 42 | 45 |
| 1335 | 86 | 83 | ND | ND |
| 1338 | 80 | 73 | 62 | 73 |
| 1339 | 76 | 71 | 51 | 71 |
| Δ3C | EBER | CD20 | EBNA1 | CD20 |
| 1003 | 90 | 84 | 73 | 84 |
| 1005 | ND | ND | 77 | 75 |
| 1183 | 73 | 72 | 83 | 72 |
| 1192 | 94 | 86 | 85 | 86 |
| 1194 | 32 | 39 | 91 | 90 |
| 1337 | 65 | 62 | 64 | 62 |
| 1340 | 104 | 100 | 96 | 100 |
| 1347 | 46 | 42 | ND | ND |
| 1348 | 66 | 67 | 61 | 67 |
| 1350 | 59 | 60 | ND | ND |
