# Supplementary material for: An EBNA3C-deleted Epstein-Barr virus (EBV) mutant causes B-cell lymphomas with delayed onset in a cord blood-humanized mouse model
Source: PLoS Pathog. 2018 Aug 20;14(8):e1007221. doi: 10.1371/journal.ppat.1007221 (PMC6117096; doi:10.1371/journal.ppat.1007221)
Supplement: S4 Table — CDR3 sequences of TCRB transcripts were deducted from RNA-seq analysis as described in the methods. (DOCX) [file ppat.1007221.s012.docx]

**S4 Table: TCRB CDR3 sequences from wild-type (WT) EBV and Δ3C EBV infected lymphomas**

| Tumor | V gene | CDR3 |
| --- | --- | --- |
| WT 1 | TRBV7-6*00 | CASSLAGGSYNEQFF |
| WT 1 | TRBV7-8*00 | CASSLLAGSLSYNEQFF |
| WT 1 | TRBV7-2*00 | CASRRTGEKGNTEAFF |
| WT 1 | TRBV25-1*00 | CASSTGTGGADTQYF |
| WT 1 | TRBV12-3*00 | CASSLQGPTYEQYF |
| WT 1 | TRBV5-1*00 | CASSLEPDGEQFF |
| WT 1 | TRBV28*00 | CASSFRGQPQHF |
| WT 3 | TRBV7-9*00 | CASSLGNTEAFF |
| WT 3 | TRBV6-2*00 | CASSFRQSGSNQPQHF |
| WT 3 | TRBV11-1*00 | CASSLAGSYNEQFF |
| WT 3 | TRBV19*00 | CASRLGGTEAFF |
| Δ3C 1 | TRBV28*00 | CASSPPPAGGPHEQFF |
| Δ3C 1 | TRBV6-1*00 | CASSARLAGRLGNEQFF |
| Δ3C 1 | TRBV28*00 | CASSLPQTDGFNEQFF |
| Δ3C 1 | TRBV5-1*00 | CASSLIDRGAEAFF |
| Δ3C 1 | TRBV18*00 | CASSPGLAGGGPYEQYF |
| Δ3C 1 | TRBV21-1*00 | CASSKRRGVSYNEQFF |
| Δ3C 1 | TRBV10-3*00 | CAISESGRPYNEQFF |
| Δ3C 1 | TRBV27*00 | CASSLGTGANYGYTF |
| Δ3C 1 | TRBV6-5*00 | CASSRRRGHNYGYTF |
| Δ3C 1 | TRBV10-3*00 | CAISTSGVLNEQFF |
| Δ3C 1 | TRBV11-2*00 | CASSLARGYTEAFF |
| Δ3C 1 | TRBV11-2*00 | CASSLEGLRWRYTF |
| Δ3C 1 | TRBV12-5*00 | CASGLTSYNEQFF |
| Δ3C 1 | TRBV6-5*00 | CASSYGTNYGYTF |
| Δ3C 1 | TRBV5-1*00 | CASSPRDSYEQYF |
| Δ3C 1 | TRBV20-1*00 | CSARALTSSEQFF |
| Δ3C 1 | TRBV7-2*00 | CASSFYTGELFF |
| Δ3C 1 | TRBV7-9*00 | CASSFSTDTQYF |
| Δ3C 1 | TRBV7-2*00 | CASSLYNSPLHF |
| Δ3C 1 | TRBV6-5*00 | CASSYGGEQYF |
| Δ3C 1 | TRBV6-1*00 | CASSEGGTQYF |
| Δ3C 2 | TRBV19*00 | CATEGGRSYTEAFF |
| Δ3C 2 | TRBV5-4*00 | CASRAAGGPPNEQFF |
| Δ3C 2 | TRBV6-1*00 | CASSEGRWENTEAFF |
| Δ3C 2 | TRBV29-1*00 | CSVVPLLAGGPLYNEQFF |
| Δ3C 2 | TRBV6-1*00 | CASSQGLAPYNEQFF |
| Δ3C 2 | TRBV13*00 | CASSPTSGRAYEQYF |
| Δ3C 2 | TRBV7-9*00 | CASSPQDHTGELFF |
| Δ3C 2 | TRBV19*00 | CASSLGGSPSRNEQFF |
| Δ3C 2 | TRBV28*00 | CASSPAGGGNTGELFF |
| Δ3C 2 | TRBV12-3*00 | CASNPTGSSYNEQFF |
| Δ3C 2 | TRBV12-3*00 | CASIRTVSYNEQFF |
| Δ3C 2 | TRBV7-9*00 | CASSLGHTEAFF |
